# Identification of a gene signature in cell cycle pathway for breast cancer prognosis using gene expression profiling data

**DOI:** 10.1186/1755-8794-1-39

**Published:** 2008-09-11

**Authors:** Jiangang Liu, Andrew Campen, Shuguang Huang, Sheng-Bin Peng, Xiang Ye, Mathew Palakal, A Keith Dunker, Yuni Xia, Shuyu Li

**Affiliations:** 1 Lilly Research Laboratories, Eli Lilly and Company, Indianapolis, IN 46285, USA; 2 School of Informatics, Indiana University-Purdue University Indianapolis, Indianapolis, IN 46202, USA

## Abstract

**Background:**

Numerous studies have used microarrays to identify gene signatures for predicting cancer patient clinical outcome and responses to chemotherapy. However, the potential impact of gene expression profiling in cancer diagnosis, prognosis and development of personalized treatment may not be fully exploited due to the lack of consensus gene signatures and poor understanding of the underlying molecular mechanisms.

**Methods:**

We developed a novel approach to derive gene signatures for breast cancer prognosis in the context of known biological pathways. Using unsupervised methods, cancer patients were separated into distinct groups based on gene expression patterns in one of the following pathways: apoptosis, cell cycle, angiogenesis, metastasis, p53, DNA repair, and several receptor-mediated signaling pathways including chemokines, EGF, FGF, HIF, MAP kinase, JAK and NF-κB. The survival probabilities were then compared between the patient groups to determine if differential gene expression in a specific pathway is correlated with differential survival.

**Results:**

Our results revealed expression of cell cycle genes is strongly predictive of breast cancer outcomes. We further confirmed this observation by building a cell cycle gene signature model using supervised methods. Validated in multiple independent datasets, the cell cycle gene signature is a more accurate predictor for breast cancer clinical outcome than the previously identified Amsterdam 70-gene signature that has been developed into a FDA approved clinical test MammaPrint^®^.

**Conclusion:**

Taken together, the gene expression signature model we developed from well defined pathways is not only a consistently powerful prognosticator but also mechanistically linked to cancer biology. Our approach provides an alternative to the current methodology of identifying gene expression markers for cancer prognosis and drug responses using the whole genome gene expression data.

## Background

DNA microarray technology has created a new paradigm for understanding cancer biology by simultaneous measurement of tens of thousands of genes in malignant or normal cells. Gene expression profiles have been utilized to identify gene signatures for cancer diagnosis and prognosis [1]. Motivated by the lack of accurate outcome prediction with the best clinical predictors of metastasis including lymph-node status and histological grade, numerous studies sought to utilize microarray technology in order to identify gene expression patterns that could be used to distinguish between patients who had the same stage of disease but different responses to treatment and hence different overall clinical outcomes. For example, a 70-gene expression signature, often referred to as the Amsterdam signature, was developed from gene expression profiles of 117 breast tumors and was strongly predictive of a short interval to distant metastases in patients with tumors that were lymph node negative [2]. The 70-gene signature was further validated in a follow-up study of 295 breast cancer patients [3]. These studies showed that gene-expression-based biomarkers were more powerful predictors of outcome than traditional clinical criteria. Recently, microarray-based gene expression signatures have also been developed to predict patient responses to therapeutic agents [4,5].

However, there are two major concerns among biologists and physicians regarding gene expression signatures obtained from microarray data as prognosis markers or predictors for drug responses [6]. First, gene signatures reported by different studies have little overlap. For example, a subset of 64 genes was identified from gene expression profiling data of 159 population-derived breast cancer patients to give an optimal separation of patients with good and poor outcomes [7]. Only three of the 64 genes were among the 70-gene prognosis signature [2]. In another study, a 76-gene signature was developed from Affymetrix array data of 286 lymph node negative breast cancer patients for risk assessment [8]. Similarly, upon comparison of this 76-gene signature with the Amsterdam 70-gene signature, only 3 genes overlapped. There are several additional prognostic models with various number of genes derived from microarray gene expression data including the intrinsic subtype model [9-11], the wound response model [12], the recurrence score model [13] and the two-gene-ratio model [14]. The gene overlap between these models is minimal. Fan and colleagues compared five models in a single dataset and found four of the five models to be concordant in their outcome prediction [15]. While this result suggested that different prognostic gene signatures may track a common set of biological characteristics, the question remains that why there is a lack of consensus gene expression models for prognosis. The van't Veer dataset, for which the 70-gene signature was derived from [2], was analyzed retrospectively [16]. It was found that different genes can be identified as prognosis markers depending on which subset of patient samples is selected as the training dataset [16], further casting the doubt on the current methodology of developing prognostic gene signatures from the whole genome transcription profiles. Second, the gene expression signatures for prognosis or drug responses are often difficult to interpret with respect to the underlying biology. Up to 30% of the signature genes have unknown function while the rest of them are associated with various unrelated biological pathways. Ultimately, finding gene signatures that can be linked to the molecular mechanisms of cancer development is critical for translating these markers into the clinic. Recent controversy in deriving gene expression patterns from microarray data to predict whether tumors will respond to chemotherapy [17] is a reflection of these two issues.

In this report, we attempted to address the above-mentioned two issues by developing a novel approach to identify gene signatures for cancer prognosis in the context of known biological pathways. Due to the nature of high dimensional data spaces in microarray studies where the number of measurements (> 10,000 mRNA transcripts) is greatly higher than the number of samples, data over-fitting is an inevitable issue [18]. Therefore, our rationale was if we attempt to identify gene signatures within well defined pathways, not only does this approach alleviate the dimensionality problem, but the mechanism-based gene signatures should also be more biologically relevant than the signatures derived from the entire human transcriptome. Unsupervised hierarchical clustering analysis was first used to divide cancer patients into separate groups based on expression patterns of genes in a known pathway. Patient survival in the different groups was then compared. If a specific pathway plays a critical role in tumor progression and metastasis, patients with distinct gene expression patterns in the pathway may have very different clinical outcomes. The results presented here indicate that the pattern of gene expression in the cell cycle pathway can indeed serve as a powerful biomarker for breast cancer prognosis. We further built a predictive model for prognosis based on the cell cycle gene signature and found our model to be more accurate than the Amsterdam 70-gene signature when tested with multiple gene expression datasets generated from several patient populations.

## Methods

### Data source

Five different gene expression profiling datasets on breast cancers were analyzed in this study. Multiple datasets were used to demonstrate repeatability of the analysis. Specific details on each dataset are summarized in Table 1. For each gene expression dataset, 20 molecular pathways were analyzed. The 20 pathways were assembled from the Ingenuity Pathway databases  and the SuperArray cancer pathway array annotations . The list of 20 pathways and genes within each pathway are provided in additional files [see additional file 1].

**Table 1:** Breast cancer gene expression profiling datasets analyzed in this study.

Reference	Study summary	Sample Size	Microarray platforms	Data download	How dataset was used in this study
Van de Vijver et al. [3]	Demonstrated that a 70-gene expression signature is a more powerful predictor for outcome than standard clinical and histological criteria in 295 primary breast cancer patients	295	Inkjet Oligo		Initial unsupervised analysis to identify outcome associated pathways.
Wang et al. [8]	Developed a 76-gene signature to predict distant metastasis using gene expression profiling data in 286 node negative primary breast cancer tumors	286	U133A		Initial unsupervised analysis to identify outcome associated pathways; Training dataset to build prognostic gene signature models.
Miller et al. [22]	Identified a 32-gene signature from 251 primary breast cancers to distinguish p53-mutant and wild-type tumors and to predict prognosis.	251	U133A		Initial unsupervised analysis to identify outcome associated pathways; Independent dataset for validating the prognostic gene signature models.
Pawitan et al. [7]	Identified a subset of 64 genes from gene expression profiles in 159 primary breast cancers that give an optimal separation of good and poor outcomes.	159	U133A		Initial unsupervised analysis to identify outcome associated pathways; Independent dataset for validating the prognostic gene signature models.
Bild et al. [21]	Developed gene expression signatures for oncogenic pathways and demonstrated these signatures are predictive of clinical outcomes in lung, breast and ovarian cancers.	171	U95Av2		Initial unsupervised analysis to identify outcome associated pathways.

### Data preprocessing

For each array study based on Affymetrix oligonucleotide platforms, we downloaded the .CEL files and generated gene expression values using the Affymetrix MAS5 algorithm with trimmed mean values normalized to 500. A trimmed mean is the average value after removing the lowest 2% and the highest 2% of all expression values on the array. Prior to analysis, each data set was preprocessed with a log_2 _transformation and subsequently expression of each gene was standardized using median-centering. Data transformation and standardization were performed using scripts written in the R statistical programming language. When a gene is represented by multiple probe sets on Affymetrix oligonucleotide arrays, the average expression value was used for further analysis.

### Hierarchical Clustering

Each pathway specific data set was analyzed by hierarchical average-linkage clustering. The clustering was performed using Gene Cluster 3.0  or using R programs. The resulting numerical output was used by Java Treeview v1.1  to generate the associated heatmaps and clustering dendrograms.

### Kaplan-Meier Survival Analysis

In addition to gene expression data, clinical information for each primary tumor sample is provided by the authors in each array study we analyzed (Table 1). The clinical data included survival and/or relapse time and censoring status. Using the available clinical outcome data, Kaplan-Meier analysis was performed on the patient groups defined by the hierarchical clustering analysis. An outcome curve for each cluster was produced using GraphPad Prism 4. The associated p-values generated from log-rank test in Kaplan-Meier analysis was used to represent the statistical significance of differential survival probabilities between the two patient groups.

### Supervised learning analysis

The PAM (Prediction Analysis for Microarray) algorithm [19] was used as the classification method. The analysis was implemented in the R programming language. A 10-fold cross validation was used by dividing the training dataset into 10 approximately equal-sized groups. The model was fitted on the 90% of the samples and tested on the remaining 10%. The procedure was repeated 10 times so each of the 10 groups was used as the testing samples and contributed to the overall error rate. The amount of shrinkage was chosen to minimize the error rate.

## Results

### Gene expression profiling datasets and the analyzed pathways

Although there are dozens of breast cancer microarray studies, the available datasets that we could utilize in our study are limited. First, to ensure statistical power, we selected datasets with at least 100 patient samples. In addition, both gene expression data and patient clinical data such as survival time and status needed to be available. To obviate fundamental difference inherent in different array platforms, we focused mainly on gene expression data based on Affymetrix oligonucleotide arrays, particularly more advanced platforms such as U95Av2 or U133 series. We also included the 295-sample dataset that served as the basis for the development and validation of the original Amsterdam 70-gene prognostic signature [3]. As indicated in Table 1, five datasets on primary breast tumors were analyzed.

The datasets in Table 1 were analyzed using 20 molecular pathways that were compiled from Ingenuity Pathway databases  and the SuperArray cancer pathway array annotations . These pathways are involved in cancer development by directly regulating angiogenesis or metastasis processes, by regulating cell cycle, apoptosis, DNA repair, or by mediating cell signaling events (Table 2). The genes in each pathway were assembled manually from literature information as of February 2007. In addition, we included the Amsterdam 70-gene signature as a control in our analysis. We also included a breast cancer gene set that contains 264 genes as known molecular markers in the prognosis and diagnosis of breast cancer. These genes were derived from literature as well as from previous microarray studies [2,3,13,20]. The 70-gene signature is a subset of the 264 breast cancer gene model. Listed in additional file 1 are the pathway names and genes associated with each pathway [see additional file 1].

**Table 2:** Gene expression in specific pathways as prognosis markers.

	Dataset				
	
Pathways	Van de Vijver [3]	Wang [8]	Miller [22]	Pawitan [7]	Bild [21]
The 70-gene signature	5.1E-07*	0.0059*	0.00020*	0.00049*	0.038*
Angiogenesis	0.069	0.30	0.12	0.0023*	0.711
Apoptosis	0.50	0.23	0.0017*	0.19	0.055
Breast cancer	3.2E-08*	0.0035*	2.4E-04*	4.7E-05*	0.050*
Chemokines	0.16	0.28	0.064	0.00045*	0.64
Cell Cycle	9.9E-09*	0.0035*	0.0017*	9.5E-05*	0.037*
DNA damage	2.2E-05*	0.055	0.036*	0.0062*	0.2
EGF	3.5E-06*	0.25	0.0049*	0.00099*	0.013*
FGF	4.9E-06*	0.033*	0.0047*	2.1E-06*	0.14
G1_S	0.0014*	0.00098*	0.0037*	0.0027*	0.21
G2_M	0.10	0.080	3.5E-04*	0.016*	0.19
HIF	0.0035*	0.030*	0.19	0.44	0.011*
JAK	0.67	0.37	0.061	0.084	0.029*
MAPK	0.0069*	0.94	0.0059*	0.25	0.76
Metastasis	0.35	0.015*	2.9E-04*	0.00037*	0.44
NER	0.92	0.80	0.27	0.16	0.64
NF-κB	0.88	0.91	0.49	0.47	0.11
p38	0.078	0.35	0.84	0.054	0.077
p53	9.2E-06*	0.066	0.0065*	5.9E-06*	0.013*
DNA Repair	1.7E-08*	0.0076*	0.047*	0.023*	0.22
Cell surface signaling	0.045*	0.13	0.025*	4.9E-05*	0.55

The numbers represent the log-rank test P values in Kaplan-Meier analysis in two patient groups defined by hierarchical clustering. *P < 0.05.

### Overall analysis strategy

**Figure 1:**
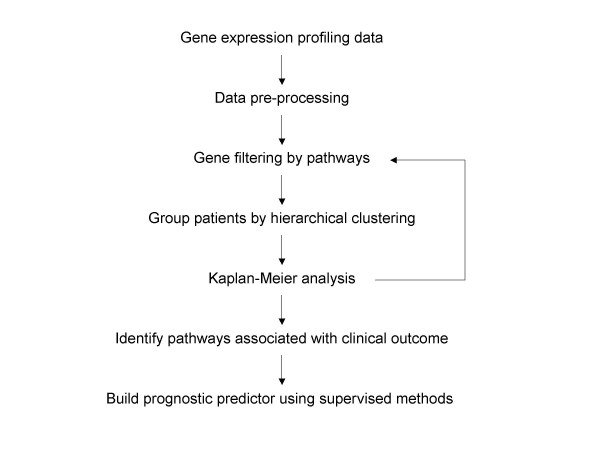
Analysis strategy. Hierarchical clustering using gene expression in specific pathways followed by Kaplan-Meier survival analysis. The pathways exhibiting strong correlation between gene expression and clinical outcome were further examined using supervised methods to build predict models.

Illustrated in Figure 1 is a flow chart describing the overall analysis. For each dataset, we first extracted expression data of genes involved in a specific pathway, followed by an unsupervised two-way hierarchical clustering analysis. If the hierarchical clustering analysis resulted in several distinct patient groups, then patient outcome in these distinct groups were compared using the Kaplan-Meier analysis. Our rationale is that if a specific pathway plays a critical role in tumor progression and metastasis, patients with distinct gene expression patterns in the pathway may have very different clinical outcome. This process was repeated for each of the 20 pathways we assembled.

The five datasets in Table 1 were analyzed as demonstrated in Figure 1 for the 20 pathways. For each hierarchical clustering, cancer patients were separated into two distinct groups that Kaplan-Meier analysis was applied to. Summarized in Table 2 are the log-rank test P values of the Kaplan-Meier survival analysis. A P-value of less than 0.05 suggests that the two patient clusters have significantly differential survival probabilities.

### Identify pathways with gene expressions correlated with clinical outcome using unsupervised clustering

**Figure 2:**
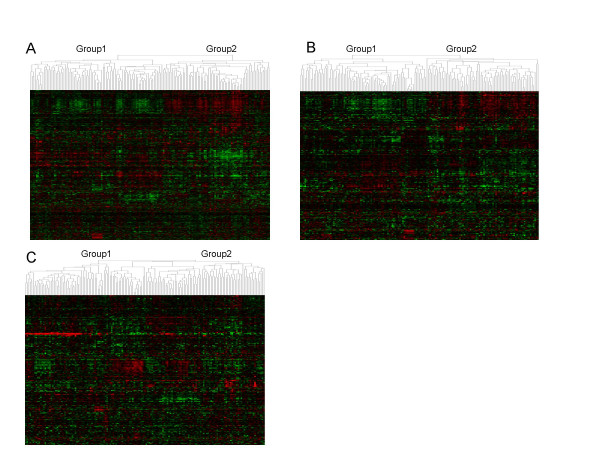
Hierarchical clustering heatmap of breast cancers based on expression of genes in breast cancer gene marker set (A), cell cycle pathway (B), and NF-κB pathway (C). The dendrograms indicated that patients are clustered into two groups (Group1 and Group2) according to their expression patterns of the specified gene set.

**Figure 3:**
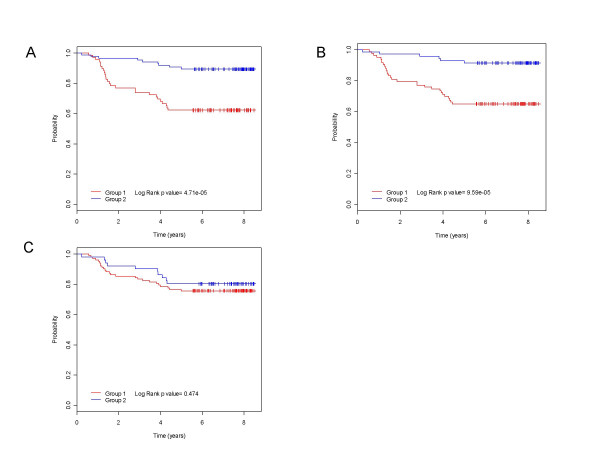
Kaplan-Meier survival analysis of breast cancer patient groups defined by the hierarchical clustering analysis shown in Figure 2 for breast cancer gene marker set (A), cell cycle pathway (B), and NF-κB pathway (C).

We first tested the Amsterdam 70-gene signature and the breast cancer gene set including 264 genes as known molecular markers in the prognosis and diagnosis of breast cancer. Our goal was to examine if patients with differential expression patterns of these markers exhibited distinct survival probabilities as one would expect. This is a proof-of-concept test and served as the positive control in our study. As demonstrated in Table 2, there is indeed a significant difference in clinical outcome between the two patient groups with distinct expression patterns of genes in the 70-gene signature or in the 264 breast cancer gene set. This result is reproducible in all of the five datasets (P < 0.05). We would like to emphasize that the five array datasets we analyzed were generated from different patient cohorts that included a total of 1,162 breast tumor samples. Figure 2A depicts a heatmap of the breast cancer gene marker expressions in 159 samples of one dataset [7]. The column dendrogram revealed these 159 patients were clustered into two groups with opposite expression patterns. The two groups exhibited a markedly different survival as revealed by the Kaplan-Meier analysis (Figure 3A).

We next investigated if gene sets based on any of the well known pathways [see additional file 1] could be used as cancer prognosis markers. As shown in Table 2, breast cancer patients with differential gene expressions in cell cycle had significantly different clinical outcome shown in all of the five datasets (P < 0.05), suggesting that the cell cycle pathway may be functionally important in breast cancer progression and that the genes in this pathway could be used as prognosis markers. EGF, FGF, G1-S and p53 pathways exhibited significant correlation between gene expression and survival in 4 datasets. This is somewhat expected given that G1-S transition is a part of the cell cycle pathway and significant roles of EGF, FGF and p53 pathway genes in regulating cell cycle. Figure 2B illustrates in one breast cancer array study [7], tumor samples can be separated into two groups with distinct expression patterns of cell cycle genes, and the two groups had significantly different survival probabilities (Figure 3B). In contrast, patients with distinct expression patterns of genes in the NF-κB pathway (Figure 2C) have similar outcomes (Figure 3C).

### Confirm prognostic gene signatures in cell cycle pathway using supervised classification

Next we applied the PAM (Prediction Analysis for Microarray) method [19], a supervised learning algorithm to confirm the predictive powers of cell cycle pathway genes for breast cancer clinical outcome, and to build a gene signature prognostic model. We used the Wang study [8] as the training dataset to build a classification model from the Amsterdam 70-gene set, the breast cancer marker gene set and the cell cycle pathway gene set, respectively, using the PAM algorithm. The models were fitted on 90% of the samples and tested on the remaining 10%. Each patient in the 10% testing samples was classified into the good or the poor prognosis groups based on the model developed using the training data. The procedure was repeated 10 times so each of the 10 groups was used as the testing samples and contributed to the overall prediction accuracy. Kaplan-Meier analysis of the predicted good and poor prognostic groups was performed to assess the predictive power of the models. We further carried out independent validation in two other datasets based on the same Affymetrix array platforms U133A (Table 1). The van de Vijver dataset [3] and the Bild dataset [21] were based on completely different microarray platforms, an InkJet oligonucleotide array and Affymetrix U95Av2 array respectively, and therefore were omitted in independent validation analysis due to technical reasons (for example, many genes in the prognostic models built on the Affymetrix U133A arrays are not represented on the InkJet oligonucleotide array and Affymetrix U95Av2 array). The patient samples in the two validation datasets [7,22] were classified into the good and poor prognostic groups respectively using the models developed from the Wang study [8], subsequently followed by Kaplan-Meier analysis. The significance of differential survival probabilities between the two groups, represented by log-rank test P values in the Kaplan-Meier analysis, were recorded as shown in Table 3. Both the cell cycle signature we developed and the previously identified breast cancer gene signature performed well as prognostic biomarkers in the training dataset and two independent validation datasets. However, the 70-gene Amsterdam signature was less accurate, particularly when evaluated using independent datasets. A set of 26 gene transcripts in the cell cycle pathway exhibited expression elevations greater than 2 fold in the poor prognosis groups in our training dataset (Table 4) and most of these genes have well documented roles in cancer development.

**Table 3:** Evaluation of cell cycle gene expression signature as breast cancer prognosis markers by supervised methods.

		Dataset		
		
Gene signature model	Number of genes used in the classification model	Training and testing: Wang dataset [8]	Independent validation: Miller dataset [22]	Independent validation: Pawitan dataset [7]
The 70-gene signature	51	6.1E-05	0.057	0.051
Breast cancer	232	2.6E-09	0.0012	0.0019
Cell Cycle	108	1.4E-06	0.0050	0.0046
Random	232	1.8E-13	0.14	0.52

The numbers represent the log-rank test P values in Kaplan-Meier analysis in the good and poor prognosis groups predicted by the Amsterdam 70-gene signature, the breast cancer gene set, the cell cycle gene set classifier, and the randomly selected gene set respectively.

**Table 4:** Expression of cell cycle genes in breast cancers.

Symbol	ID	Fold	Description
BIRC5	332	4.25	Baculoviral IAP repeat-containing 5, antiapoptotic cell cycle regulator, expression in many cancers is associated with poor prognosis and mediates cancer cell resistance to taxol and radiation; rat Birc5 is upregulated in response to acute pancreatitis
BRCA2	675	2.13	Breast cancer 2 early onset, a transcription coactivator that binds to RAD51 and TP53, regulates cell proliferation, cell cycle progression, and DNA repair; mutations in the corresponding gene are associated with Fanconi anemia and multiple cancers
CCNA2	890	3.11	Cyclin A2, a cyclin-dependent protein kinase regulator, promotes G2/M transition, progression through cell cycle, cell proliferation, and phosphorylation of proteins; upregulated in male germ cell tumors and testicular tumors
CCNB1	891	2.43	Cyclin B1, complexes with CDC2 to promote nuclear membrane and Golgi disassembly, chromosome condensation, and microtubule reorganization, aberrant expression is associated with multiple neoplasms, increased expression correlates with Alzheimer disease
CCNB2	9133	3.28	Cyclin B2, a CDC2 kinase-associated cyclin that is involved in Golgi apparatus disassembly, may function in p53 (TP53)-mediated cell cycle arrest at the G2/M transition, may mediate cell cycle arrest and is overexpressed in nonendometrioid carcinomas
CCNE1	898	3.01	Cyclin E1, a CDK and histone deacetylase regulator, regulates mitotic G1-S phase transition and promotes cell proliferation, involved in peptidyl-threonine phosphorylation and aging, aberrant mRNA and protein expression is associated with several cancers
CCNE2	9134	2.75	Cyclin E2, a cyclin-dependent protein kinase regulator that binds CDK2 and CDK3, regulates cell cycle checkpoint; mRNA upregulation correlates with breast and lung cancer, mouse Ccne2 is overexpressed in TPA-induced carcinomas and fore stomach cancers
CDC2	983	2.87	Cell division cycle control protein 2, a cyclin-dependent protein kinase that acts in DNA damage checkpoint, inhibits apoptosis and EGFR signaling, expression is increased in Alzheimer disease, viremia associated with HIV infection, and various cancers
CDC20	991	3.72	Cell division cycle 20, a mitotic checkpoint protein and transcriptional repressor, activates the mitotically phosphorylated form of the anaphase promoting complex as well as the mitotic spindle checkpoint, overexpressed in gastric cancer
CDC25A	993	2.7	Cell division cycle 25A, protein tyrosine-threonine phosphatase, regulates G1-S and G2-M phase transitions, functions in apoptosis and oxidative stress response, activity increases in Alzheimer's disease neurons, overexpressed in many cancers
CDC45L	8318	4.9	Cell division cycle 45 like, associates with ORC2L, MCM7, and POLA2, predicted to be involved in the initiation of DNA replication; corresponding gene is located in a chromosomal region frequently deleted in DiGeorge syndrome
CDC6	990	2.47	Cell division cycle 6, involved in DNA replication initiation, may regulate DNA licensing, pre-replicative complex formation and cell proliferation, upregulated in cervical intraepithelial neoplasia and cervical cancer, downregulated in prostate cancer
CDKN2A	1029	2.13	Cyclin dependent kinase inhibitor 2A, interacts with CDK4 and CDK6, involved in aging, anoikis, and cell cycle arrest, regulates transcription factor activity and cell proliferation, aberrantly expressed in psoriasis and several types of cancer
CHEK1	1111	2.54	Checkpoint homolog 1 (S. pombe), protein kinase, required for mitotic G2 checkpoint in response to radition-induced DNA damage, inhibits mitotic entry after DNA damage via mechanism involving CDC25, alternative form is associated with lung cancer
CKS1B	1163	2.08	CDC28 protein kinase regulatory subunit 1B, essential for SKP2-mediated ubiquitination of CDKN1A and CDKN1B, regulate cell cycle progression, aberrant protein expression is associated with several cancers
CKS2	1164	2.27	CDC28 protein kinase regulatory subunit 2, a protein that binds p34 CDC2 and may regulate cell cycle progression, upregulated in pancreatic cancer cell lines
E2F1	1869	2.39	E2F transcription factor 1, inhibits cell proliferation, aberrant expression correlates with several neoplasms and Alzheimer disease associated with Down syndrome; knockout of mouse E2f1 is associated with early onset of diabetes and Sjogren's syndrome
GTSE1	51512	2.61	G-2 and S-phase expressed 1, a cell cycle-regulated and microtubule-associated protein that acts in nuclear-cytoplasmic shuttling of p53 (TP53), may play a role in DNA-damage induced apoptosis through regulation of p53 function during S and G(2) phases
KPNA2	3838	2.18	Karyopherin alpha 2, an NLS binding protein that acts in the nuclear transport of proteins and may play a role in V(D)J recombination, upregulated in breast cancer; human KPNA2 gene map position correlates with fetal growth retardation
MAD2L1	4085	3	MAD2 mitotic arrest deficient-like 1 (yeast), mitotic spindle checkpoint complex component, inhibits anaphase-promoting complex activation, binds MAD1L1, altered expression is linked to several cancers and adult T-cell leukemia
MCM2	4171	2.88	Mini chromosome maintenance deficient 2, binds chromatin, regulates the onset of DNA replication, inhibits the helicase activity of the MCM 4,6,7 complex, expression is altered and is prognostic in a number of cancers
MCM4	4173	2.82	Minichromosome maintenance deficient 4, forms a single stranded ATP-dependent DNA helicase with MCM6 and MCM7, may monitor sites of unreplicated DNA, displacement from replicated chromatin may ensure that DNA is only replicated once per cell cycle
MCM5	4174	2.39	Mini chromosome maintenance deficient 5, transcriptional coactivator that interacts with STAT1, enhances IFNG -induced and STAT1 -dependent transactivation, localizes to unreplicated chromatin, upregulated in anaplastic thyroid carcinoma
MCM6	4175	2.15	MCM6 minichromosome maintenance deficient 6, a component of the heterohexameric MCM complex that has ATP-dependent DNA helicase activity, acts in DNA replication initiation, upregulated in mantle cell lymphoma
MKI67	4288	2.43	Ki-67 antigen, induces chromatin compaction, acts in cell proliferation, expression is altered in neoplasms including osteosarcoma and prostate, breast and esophageal cancer; gene is mutated in cervical, colon and lung carcinoma cell lines
RAD51	5888	2.13	RAD51 homolog, a DNA binding ATPase that acts in apoptosis, cell proliferation, p53-mediated DNA damage response, and double-strand break repair via homologous recombination, aberrant expression correlates with bloom syndrome and several neoplasms

The fold changes represent the ratio of expression in the poor prognosis group over that in the good prognosis group in the training dataset [8].

We also randomly selected 232 genes, the number of genes used in the breast cancer gene set signature, to build prediction models and the random models were similarly assessed in the training dataset and two independent datasets as described above. This random testing was repeated 100 times and the P-values in the Kaplan-Meier analysis were the average of the 100 experiments. Interestingly, the classification models based on randomly selected genes performed exceptionally well in the training dataset using the10-fold cross validation procedure (Table 3), suggesting if one uses a large number of genes to build a prediction model, some of the randomly chosen genes will be differentially expressed between the good and poor prognosis groups by chance and therefore provide prognostic values. However, when analyzed in independent datasets of different patient cohorts, the models with random genes did not show predictive power (Table 3), demonstrating that microarray based gene expression predictors must be tested through multiple independent datasets to validate their robustness, a practice that has failed to be recognized by most published studies in the literature.

## Discussion

Our analysis demonstrated that differential expression of genes in the cell cycle pathway is associated with differential patient outcome in breast cancers, suggesting that cell cycle regulation may be one of the most important factors contributing to breast cancer progression. In fact, cell proliferation markers have been extensively investigated for their prognostic values [23,24]. A literature search has revealed expressions of many cell cycle related genes are correlated with breast cancer progression and patient survival as individual outcome predictors. Cyclins bind and activate cyclin-dependent kinases to drive cell cycle progression. The prognostic role of cyclins has been retrospectively assessed in numerous studies. For example, measurement of cyclin E by Western Blot and immunohistochemistry in 395 breast cancer patients showed that higher level of total cyclin E is strongly correlated with poor outcome [25]. Cyclin A, B and D also appeared to be strong prognostic markers in some studies [26-28]. CDC25A is a protein tyrosine-threonine phosphatase and regulates G1-S and G2-M transitions. Overexpression of CDC25A is associated with poor prognosis in breast cancers [29]. Several independent reports demonstrated that high level E2F1 expression correlates with reduced disease-free survival in node-negative breast cancer patients [30-32]. Ki-67 (MKI67) antigen induces chromatin condensation and is a well known cell proliferation marker. A recent review summarized that Ki-67 expression assayed by IHC showed prognostic values in 15 studies where a total of more than 5000 tumor samples were analyzed [24]. While these cell cycle related genes have been individually linked to breast cancer outcome, the multi-gene signature we applied in our analysis may provide a more accurate predictor, and more importantly these genes are mechanistically implicated in breast cancer progression. A close examination of gene identities in the cell cycle pathway, the Amsterdam 70-gene signature, and the control breast cancer gene signature revealed that the Amsterdam signature only included one cell cycle gene (cyclin E2). In contrast, the 232-gene breast cancer signature and the 108-gene cell cycle pathway have a 25-gene overlap including several cyclins (cyclin B1, B2, D1, E1, E2), cyclin-dependent kinases (CDK2, CDK4), tumor suppressors p53 and RB1, and the proliferation marker Ki-67, suggesting that predictive power of the control breast cancer signature may be due to the presence of these cell cycle related genes.

Adjuvant therapy and hormonal treatment of breast cancer patients have been demonstrated to improve survival. However, these treatment regimens are costly and could have serious side effects, therefore, should only be recommended to high risk patients. Traditional prognostic factors such as lymph node status, tumor diameter and histological grades do not accurately predict clinical behaviors of the breast tumors and as a result, patients can be over-treated or under-treated depending on the clinicpathological guidelines. Identification of additional prognostic markers is important for clinicians to select the most appropriate systemic treatments for individual patients according to their risks of relapse or death. Cell proliferation is a key feature of breast tumor progression and has been widely evaluated as a prognosis factor. Although many proliferation markers have been established as robust prognosticators, they have not been applied in clinic due to various technical barriers. For example, 3H-thymidine labeling index (TLI) was one of the first methods developed to evaluate proliferative activity through measuring 3H-thymidine uptake by tumor cells undergoing DNA synthesis [33-35]. However, it has never been adopted as a standard prognostic marker because the experiment requires fresh tumor tissue and a complex and time consuming radioactive assay for *in vivo *administration of labeled substances. Measurement of DNA content by flow cytometry has provided a reliable approach to determine tumor cell proliferative activity represented by S-phase fraction (SPF) [36], but the lack of standardized procedure to prepare and analyze tumor samples precluded use of this method as a routine assay [37]. Application of proliferation antigen Ki-67 is hampered as the Ki-67 monoclonal antibody could only be used on fresh or frozen tissue since fixation greatly reduced immunostaining [38]. The predictive power of abovementioned cell cycle regulators such as cyclins has not yet proved definitive since in some studies the correlation between protein level and clinical outcome is not significant [23]. The Amsterdam 70-gene expression signature as breast cancer prognosis marker has been validated in follow-up studies [39,40], and a clinical assay MammaPrint^® ^has recently been cleared by FDA. However, the two issues associated with the current gene expression signature markers for prognosis, i.e. the lack of a consensus gene set and the difficulty to understand underlying mechanisms, may prevent them from being widely accepted. The cell cycle gene signature we identified in this study has provided a prognostic gene expression marker that not only performed better than the Amsterdam 70-gene signature but is also mechanistically linked to breast cancer progression.

There have been recent reports to incorporate biological pathway information into classification models by using a network analysis approach [41] or to identify functional gene sets from various sources including Gene Ontology to distinguish two different biological phenotypes [42,43]. In this study, we assembled 20 pathways that are known to be involved in cancer development and progression, and then extracted expression data of genes only in these pathways in order to identify a mechanistic gene signature biomarker for breast cancer prognosis. We first selected pathways according to their classification powers based on unsupervised analysis, followed by building prognostic gene signature models using the standard supervised methods. The signature developed after pre-selecting relevant pathways should be more reliable and generally applicable as demonstrated by our validation when applied to multiple independent datasets. This is not surprising since the signature is derived from the cell cycle pathway and it has been well documented that cell cycle control plays a critical role in determining breast cancer outcomes.

We also recognize the limitation of our study. While the cell cycle gene signature derived from a training dataset [8] performed well in prognosis prediction in two independent validation datasets [7,22], we did not specifically examine how stable the signature is by building multiple signatures in different datasets in the context of cell cycle pathway and then comparing these signatures for the extent of overlap. We reasoned that there could be significant overlap simply due to a much smaller gene set that we started with in signature model building. Furthermore, we did not attempt to understand the cell cycle signature at the individual gene level to interpret the role of each gene in disease progression based on the numerical coefficients in the signature model because these numerical parameters are heavily impacted by technical variations. Nevertheless, our pathway oriented approach and the analysis results strongly suggest a critical role of the cell cycle pathway in breast cancer progression, which is also consistent with what has been known from a rich collection of literature information.

## Conclusion

Post-genomic technologies have provided a new paradigm in developing tailored therapeutic strategies for treating complex diseases. One notable example is the development of gene expression signatures based on microarray data to predict prognosis and responses to chemotherapy in cancers [5]. Several studies have revealed that multiplex gene expression markers are more powerful in predicting clinical outcomes than the traditional clinical criteria. However, the promise of applying these gene signature biomarkers in clinic is hampered because the underlying biology of gene signatures in cancer development is not well understood. Furthermore, different studies often report different gene expression predictors for the same cancer type and as a result, many biologists and physicians remain skeptical of the gene signature concept. In this study, we developed a novel approach to derive gene expression signatures for cancer prognosis in the context of known biological pathways. Our analysis not only generated mechanism based gene signature predictors, but also shed light on the role of different molecular pathways in cancer development. To our knowledge, the current study is the first effort to integrate gene expression profiling data and well known pathway information to develop pathway specific gene expression signatures for cancer prognosis, and our approach will likely provide a new direction in the Oncogenomics field to develop gene signature biomarkers. The predictive power of the cell cycle gene signature for breast cancer prognosis as demonstrated in the present study warrants further investigation such as prospective clinical trials to explore its utility in clinic. Moreover, the methodology we developed could be utilized to identify gene signature biomarkers to guide clinical development of novel cancer therapeutic agents.

## Note Added in Proof

While this manuscript was in preparation, using a completely different approach, Mosley and Keri described a similar observation that cell cycle genes dictate the power of breast cancer prognostic gene list [44].

## Competing interests

The authors declare that they have no competing interests.

## Availability and requirements 

http://bonsai.ims.u-tokyo.ac.jp/~mdehoon/software/cluster/ :  providing open source clustering software.

http://jtreeview.sourceforge.net/ :  providing open source Java TreeView software.

http://www.superarray.com/home.php  :  providing pathway focused arrays. 

http://www.ingenuity.com/  : providing pathway analysis tools for intepretation of genomics data.   

## Authors' contributions

YX and SL designed study. JL, AC, and SH performed analysis. SBP, XY, MP, AKD, YX and SL interpreted results. SL wrote the manuscript. SBP, XY, MP, AKD, and YX revised the manuscript. All authors read and approved the final manuscript.

## Pre-publication history

The pre-publication history for this paper can be accessed here:



## Supplementary Material

Additional file 1 Pathways and associated genes analyzed in the study. Click here for file

## References

[B1] Quackenbush J (2006). Microarray analysis and tumor classification. N Engl J Med.

[B2] van't Veer LJ, Dai H, Vijver MJ van de, He YD, Hart AA, Mao M, Peterse HL, Kooy K van der, Marton MJ, Witteveen AT, Schreiber GJ, Kerkhoven RM, Roberts C, Linsley PS, Bernards R, Friend SH (2002). Gene expression profiling predicts clinical outcome of breast cancer. Nature.

[B3] Vijver MJ van de, He YD, van't Veer LJ, Dai H, Hart AA, Voskuil DW, Schreiber GJ, Peterse JL, Roberts C, Marton MJ, Parrish M, Atsma D, Witteveen A, Glas A, Delahaye L, Velde T van der, Bartelink H, Rodenhuis S, Rutgers ET, Friend SH, Bernards R (2002). A gene-expression signature as a predictor of survival in breast cancer. N Engl J Med.

[B4] Lee JK, Havaleshko DM, Cho H, Weinstein JN, Kaldjian EP, Karpovich J, Grimshaw A, Theodorescu D (2007). A strategy for predicting the chemosensitivity of human cancers and its application to drug discovery. Proc Natl Acad Sci USA.

[B5] Potti A, Dressman HK, Bild A, Riedel RF, Chan G, Sayer R, Cragun J, Cottrill H, Kelley MJ, Petersen R, Harpole D, Marks J, Berchuck A, Ginsburg GS, Febbo P, Lancaster J, Nevins JR (2006). Genomic signatures to guide the use of chemotherapeutics. Nat Med.

[B6] Massague J (2007). Sorting out breast-cancer gene signatures. N Engl J Med.

[B7] Pawitan Y, Bjohle J, Amler L, Borg AL, Egyhazi S, Hall P, Han X, Holmberg L, Huang F, Klaar S, Liu ET, Miller L, Nordgren H, Ploner A, Sandelin K, Shaw PM, Smeds J, Skoog L, Wedren S, Bergh J (2005). Gene expression profiling spares early breast cancer patients from adjuvant therapy: derived and validated in two population-based cohorts. Breast Cancer Res.

[B8] Wang Y, Klijn JG, Zhang Y, Sieuwerts AM, Look MP, Yang F, Talantov D, Timmermans M, Meijer-van Gelder ME, Yu J, Jatkoe T, Berns EM, Atkins D, Foekens JA (2005). Gene-expression profiles to predict distant metastasis of lymph-node-negative primary breast cancer. Lancet.

[B9] Perou CM, Sorlie T, Eisen MB, Rijn M van de, Jeffrey SS, Rees CA, Pollack JR, Ross DT, Johnsen H, Akslen LA, Fluge O, Pergamenschikov A, Williams C, Zhu SX, Lonning PE, Borresen-Dale AL, Brown PO, Botstein D (2000). Molecular portraits of human breast tumours. Nature.

[B10] Sorlie T, Perou CM, Tibshirani R, Aas T, Geisler S, Johnsen H, Hastie T, Eisen MB, Rijn M van de, Jeffrey SS, Thorsen T, Quist H, Matese JC, Brown PO, Botstein D, Eystein Lonning P, Borresen-Dale AL (2001). Gene expression patterns of breast carcinomas distinguish tumor subclasses with clinical implications. Proc Natl Acad Sci USA.

[B11] Sorlie T, Tibshirani R, Parker J, Hastie T, Marron JS, Nobel A, Deng S, Johnsen H, Pesich R, Geisler S, Demeter J, Perou CM, Lonning PE, Brown PO, Borresen-Dale AL, Botstein D (2003). Repeated observation of breast tumor subtypes in independent gene expression data sets. Proc Natl Acad Sci USA.

[B12] Chang HY, Nuyten DS, Sneddon JB, Hastie T, Tibshirani R, Sorlie T, Dai H, He YD, van't Veer LJ, Bartelink H, Rijn M van de, Brown PO, Vijver MJ van de (2005). Robustness, scalability, and integration of a wound-response gene expression signature in predicting breast cancer survival. Proc Natl Acad Sci USA.

[B13] Paik S, Shak S, Tang G, Kim C, Baker J, Cronin M, Baehner FL, Walker MG, Watson D, Park T, Hiller W, Fisher ER, Wickerham DL, Bryant J, Wolmark N (2004). A multigene assay to predict recurrence of tamoxifen-treated, node-negative breast cancer. N Engl J Med.

[B14] Ma XJ, Wang Z, Ryan PD, Isakoff SJ, Barmettler A, Fuller A, Muir B, Mohapatra G, Salunga R, Tuggle JT, Tran Y, Tran D, Tassin A, Amon P, Wang W, Wang W, Enright E, Stecker K, Estepa-Sabal E, Smith B, Younger J, Balis U, Michaelson J, Bhan A, Habin K, Baer TM, Brugge J, Haber DA, Erlander MG, Sgroi DC (2004). A two-gene expression ratio predicts clinical outcome in breast cancer patients treated with tamoxifen. Cancer Cell.

[B15] Fan C, Oh DS, Wessels L, Weigelt B, Nuyten DS, Nobel AB, van't Veer LJ, Perou CM (2006). Concordance among gene-expression-based predictors for breast cancer. N Engl J Med.

[B16] Ein-Dor L, Kela I, Getz G, Givol D, Domany E (2005). Outcome signature genes in breast cancer: is there a unique set?. Bioinformatics.

[B17] Coombes KR, Wang J, Baggerly KA (2007). Microarrays: retracing steps. Nat Med.

[B18] Clarke R, Ressom HW, Wang A, Xuan J, Liu MC, Gehan EA, Wang Y (2008). The properties of high-dimensional data spaces: implications for exploring gene and protein expression data. Nat Rev Cancer.

[B19] Tibshirani R, Hastie T, Narasimhan B, Chu G (2002). Diagnosis of multiple cancer types by shrunken centroids of gene expression. Proc Natl Acad Sci USA.

[B20] Hu Y, Hines LM, Weng H, Zuo D, Rivera M, Richardson A, LaBaer J (2003). Analysis of genomic and proteomic data using advanced literature mining. J Proteome Res.

[B21] Bild AH, Yao G, Chang JT, Wang Q, Potti A, Chasse D, Joshi MB, Harpole D, Lancaster JM, Berchuck A, Olson JA, Marks JR, Dressman HK, West M, Nevins JR (2006). Oncogenic pathway signatures in human cancers as a guide to targeted therapies. Nature.

[B22] Miller LD, Smeds J, George J, Vega VB, Vergara L, Ploner A, Pawitan Y, Hall P, Klaar S, Liu ET, Bergh J (2005). An expression signature for p53 status in human breast cancer predicts mutation status, transcriptional effects, and patient survival. Proc Natl Acad Sci USA.

[B23] Colozza M, Azambuja E, Cardoso F, Sotiriou C, Larsimont D, Piccart MJ (2005). Proliferative markers as prognostic and predictive tools in early breast cancer: where are we now?. Ann Oncol.

[B24] van Diest PJ, Wall E van der, Baak JP (2004). Prognostic value of proliferation in invasive breast cancer: a review. J Clin Pathol.

[B25] Keyomarsi K, Tucker SL, Buchholz TA, Callister M, Ding Y, Hortobagyi GN, Bedrosian I, Knickerbocker C, Toyofuku W, Lowe M, Herliczek TW, Bacus SS (2002). Cyclin E and survival in patients with breast cancer. N Engl J Med.

[B26] Kuhling H, Alm P, Olsson H, Ferno M, Baldetorp B, Parwaresch R, Rudolph P (2003). Expression of cyclins E, A, and B, and prognosis in lymph node-negative breast cancer. J Pathol.

[B27] Peters MG, Vidal Mdel C, Gimenez L, Mauro L, Armanasco E, Cresta C, Bal de Kier Joffe E, Puricelli L (2004). Prognostic value of cell cycle regulator molecules in surgically resected stage I and II breast cancer. Oncol Rep.

[B28] Suzuki T, Urano T, Miki Y, Moriya T, Akahira J, Ishida T, Horie K, Inoue S, Sasano H (2007). Nuclear cyclin B1 in human breast carcinoma as a potent prognostic factor. Cancer Sci.

[B29] Evans KL (2000). Overexpression of CDC25A associated with poor prognosis in breast cancer. Mol Med Today.

[B30] Baldini E, Camerini A, Sgambato A, Prochilo T, Capodanno A, Pasqualetti F, Orlandini C, Resta L, Bevilacqua G, Collecchi P (2006). Cyclin A and E2F1 overexpression correlate with reduced disease-free survival in node-negative breast cancer patients. Anticancer Res.

[B31] Han S, Park K, Bae BN, Kim KH, Kim HJ, Kim YD, Kim HY (2003). E2F1 expression is related with the poor survival of lymph node-positive breast cancer patients treated with fluorouracil, doxorubicin and cyclophosphamide. Breast Cancer Res Treat.

[B32] Vuaroqueaux V, Urban P, Labuhn M, Delorenzi M, Wirapati P, Benz CC, Flury R, Dieterich H, Spyratos F, Eppenberger U, Eppenberger-Castori S (2007). Low E2F1 transcript levels are a strong determinant of favorable breast cancer outcome. Breast Cancer Res.

[B33] Lloveras B, Edgerton S, Thor AD (1991). Evaluation of in vitro bromodeoxyuridine labeling of breast carcinomas with the use of a commercial kit. Am J Clin Pathol.

[B34] Meyer JS, Connor RE (1977). In vitro labeling of solid tissues with tritiated thymidine for autoradiographic detection of S-phase nuclei. Stain Technol.

[B35] Waldman FM, Chew K, Ljung BM, Goodson W, Hom J, Duarte LA, Smith HS, Mayall B (1991). A comparison between bromodeoxyuridine and 3H thymidine labeling in human breast tumors. Mod Pathol.

[B36] Hedley DW, Rugg CA, Gelber RD (1987). Association of DNA index and S-phase fraction with prognosis of nodes positive early breast cancer. Cancer Res.

[B37] Baldetorp B, Bendahl PO, Ferno M, Alanen K, Delle U, Falkmer U, Hansson-Aggesjo B, Hockenstrom T, Lindgren A, Mossberg L (1995). Reproducibility in DNA flow cytometric analysis of breast cancer: comparison of 12 laboratories' results for 67 sample homogenates. Cytometry.

[B38] Urruticoechea A, Smith IE, Dowsett M (2005). Proliferation marker Ki-67 in early breast cancer. J Clin Oncol.

[B39] Bueno-de-Mesquita JM, van Harten WH, Retel VP, van't Veer LJ, van Dam FS, Karsenberg K, Douma KF, van Tinteren H, Peterse JL, Wesseling J, Wu TS, Atsma D, Rutgers EJ, Brink G, Floore AN, Glas AM, Roumen RM, Bellot FE, van Krimpen C, Rodenhuis S, Vijver MJ van de, Linn SC (2007). Use of 70-gene signature to predict prognosis of patients with node-negative breast cancer: a prospective community-based feasibility study (RASTER). Lancet Oncol.

[B40] Buyse M, Loi S, van't Veer L, Viale G, Delorenzi M, Glas AM, d'Assignies MS, Bergh J, Lidereau R, Ellis P, Harris A, Bogaerts J, Therasse P, Floore A, Amakrane M, Piette F, Rutgers E, Sotiriou C, Cardoso F, Piccart MJ (2006). Validation and clinical utility of a 70-gene prognostic signature for women with node-negative breast cancer. J Natl Cancer Inst.

[B41] Chuang HY, Lee E, Liu YT, Lee D, Ideker T (2007). Network-based classification of breast cancer metastasis. Mol Syst Biol.

[B42] Eichler GS, Reimers M, Kane D, Weinstein JN (2007). The LeFE algorithm: embracing the complexity of gene expression in the interpretation of microarray data. Genome Biol.

[B43] Subramanian A, Tamayo P, Mootha VK, Mukherjee S, Ebert BL, Gillette MA, Paulovich A, Pomeroy SL, Golub TR, Lander ES, Mesirov JP (2005). Gene set enrichment analysis: a knowledge-based approach for interpreting genome-wide expression profiles. Proc Natl Acad Sci USA.

[B44] Mosley JD, Keri RA (2008). Cell cycle correlated genes dictate the prognostic power of breast cancer gene lists. BMC Med Genomics.

