# Supplementary material for: Identification of a gene signature in cell cycle pathway for breast cancer prognosis using gene expression profiling data
Source: BMC Med Genomics. 2008 Sep 11;1:39. doi: 10.1186/1755-8794-1-39 (PMC2551605; doi:10.1186/1755-8794-1-39)
Supplement: Additional file 1 — Pathways and associated genes analyzed in the study. [file 1755-8794-1-39-S1.doc]

Supplemental Table 1. Pathways and associated genes analyzed in the study.

| Pathway | Gene Symbol | Entrez Gene ID |
| --- | --- | --- |
| angiogenesis | CDH5 | 31 |
| angiogenesis | HPSE | 721 |
| angiogenesis | LECT1 | 894 |
| angiogenesis | NUDT6 | 981 |
| angiogenesis | COL4A3 | 1335 |
| angiogenesis | CSF3 | 1511 |
| angiogenesis | JAG1 | 1913 |
| angiogenesis | ECGF1 | 1964 |
| angiogenesis | EDG1 | 1972 |
| angiogenesis | EFNA1 | 1995 |
| angiogenesis | EFNA2 | 1996 |
| angiogenesis | EFNA3 | 1997 |
| angiogenesis | EFNA5 | 1999 |
| angiogenesis | EFNB2 | 2001 |
| angiogenesis | EGF | 2003 |
| angiogenesis | ENG | 2097 |
| angiogenesis | EPAS1 | 2111 |
| angiogenesis | EPHB4 | 2130 |
| angiogenesis | EREG | 2149 |
| angiogenesis | AKT1 | 2150 |
| angiogenesis | F2 | 2203 |
| angiogenesis | FGF1 | 2326 |
| angiogenesis | FGF2 | 2327 |
| angiogenesis | FGF6 | 2332 |
| angiogenesis | FGFR3 | 2341 |
| angiogenesis | FIGF | 2353 |
| angiogenesis | STAB1 | 2655 |
| angiogenesis | FLT1 | 2696 |
| angiogenesis | GAPDH | 3355 |
| angiogenesis | ANGPTL3 | 3851 |
| angiogenesis | ANG | 3971 |
| angiogenesis | ANGPT1 | 4000 |
| angiogenesis | ANGPT2 | 4030 |
| angiogenesis | ANPEP | 4146 |
| angiogenesis | CXCL1 | 4228 |
| angiogenesis | CXCL2 | 4230 |
| angiogenesis | CXCL3 | 4231 |
| angiogenesis | HGF | 4475 |
| angiogenesis | HIF1A | 4494 |
| angiogenesis | ID1 | 4734 |
| angiogenesis | ID3 | 4736 |
| angiogenesis | IFNA1 | 4771 |
| angiogenesis | IFNB1 | 4791 |
| angiogenesis | IFNG | 4792 |
| angiogenesis | IGF1 | 4811 |
| angiogenesis | IL1B | 4865 |
| angiogenesis | IL6 | 4880 |
| angiogenesis | IL8 | 4886 |
| angiogenesis | IL10 | 4892 |
| angiogenesis | IL12A | 4898 |
| angiogenesis | IL18 | 4912 |
| angiogenesis | CXCL10 | 4932 |
| angiogenesis | ITGAV | 4978 |
| angiogenesis | ITGB3 | 4983 |
| angiogenesis | KDR | 5098 |
| angiogenesis | LAMA5 | 5286 |
| angiogenesis | LEP | 5329 |
| angiogenesis | MDK | 5582 |
| angiogenesis | CXCL9 | 5640 |
| angiogenesis | MMP2 | 5666 |
| angiogenesis | MMP9 | 5670 |
| angiogenesis | MMP19 | 5680 |
| angiogenesis | NOTCH4 | 6072 |
| angiogenesis | NPPB | 6087 |
| angiogenesis | NPR1 | 6090 |
| angiogenesis | ANGPTL4 | 6417 |
| angiogenesis | TNFRSF12A | 6562 |
| angiogenesis | PDGFA | 6686 |
| angiogenesis | PDGFB | 6691 |
| angiogenesis | PECAM1 | 6800 |
| angiogenesis | SERPINF1 | 6807 |
| angiogenesis | PF4 | 6837 |
| angiogenesis | PGF | 6864 |
| angiogenesis | PLAU | 6946 |
| angiogenesis | PLG | 6971 |
| angiogenesis | VEGFA | 7422 |
| angiogenesis | AGGF1 | 7549 |
| angiogenesis | STAB2 | 7861 |
| angiogenesis | B2M | 8320 |
| angiogenesis | PLXDC1 | 8494 |
| angiogenesis | PTEN | 8566 |
| angiogenesis | PTGS1 | 8604 |
| angiogenesis | PTGS2 | 8605 |
| angiogenesis | BAI1 | 8625 |
| angiogenesis | PTN | 8663 |
| angiogenesis | ACTB | 8958 |
| angiogenesis | RPS27A | 9145 |
| angiogenesis | CCL2 | 9238 |
| angiogenesis | CCL11 | 9246 |
| angiogenesis | CXCL6 | 9262 |
| angiogenesis | CXCL11 | 9263 |
| angiogenesis | CXCL5 | 9264 |
| angiogenesis | TEK | 10436 |
| angiogenesis | TGFA | 10461 |
| angiogenesis | TGFB1 | 10462 |
| angiogenesis | TGFB2 | 10464 |
| angiogenesis | TGFB3 | 10465 |
| angiogenesis | TGFBR1 | 10468 |
| angiogenesis | THBS1 | 10479 |
| angiogenesis | THBS2 | 10480 |
| angiogenesis | TIE1 | 10493 |
| angiogenesis | TIMP1 | 10494 |
| angiogenesis | TIMP2 | 10495 |
| angiogenesis | TIMP3 | 10496 |
| angiogenesis | TNF | 10539 |
| angiogenesis | TNFAIP2 | 10542 |
| angiogenesis | TNNT1 | 10552 |
| angiogenesis | VEGFB | 11123 |
| angiogenesis | VEGFC | 11124 |
| angiogenesis | COL18A1 | 12108 |
| angiogenesis | NRP2 | 12937 |
| angiogenesis | NRP1 | 12938 |
| angiogenesis | SPHK1 | 12980 |
| angiogenesis | SH2D2A | 13130 |
| angiogenesis | HAND2 | 13561 |
| angiogenesis | TNFSF15 | 14025 |
| angiogenesis | PROK2 | 60675 |
| apoptosis | CHUK | 1160 |
| apoptosis | PARP1 | 1496 |
| apoptosis | PLCG1 | 1630 |
| apoptosis | DFFA | 1810 |
| apoptosis | DFFB | 1811 |
| apoptosis | ENDOG | 2094 |
| apoptosis | ACIN1 | 2498 |
| apoptosis | IKBKB | 2852 |
| apoptosis | GAS2 | 3483 |
| apoptosis | APAF1 | 4556 |
| apoptosis | HRAS | 4634 |
| apoptosis | BIRC2 | 4649 |
| apoptosis | BIRC3 | 4660 |
| apoptosis | FAS | 4861 |
| apoptosis | FASLG | 4871 |
| apoptosis | KRAS | 5147 |
| apoptosis | LMNA | 5377 |
| apoptosis | MAP3K5 | 5603 |
| apoptosis | NFKB1 | 6020 |
| apoptosis | NFKB2 | 6021 |
| apoptosis | NFKBIA | 6022 |
| apoptosis | NFKBIB | 6023 |
| apoptosis | NRAS | 6102 |
| apoptosis | PLCG2 | 6964 |
| apoptosis | CYCS | 7069 |
| apoptosis | PRKCA | 8025 |
| apoptosis | PRKCE | 8050 |
| apoptosis | PRKCQ | 8105 |
| apoptosis | MAPK1 | 8136 |
| apoptosis | MAPK3 | 8137 |
| apoptosis | MAPK8 | 8152 |
| apoptosis | MAP2K1 | 8164 |
| apoptosis | MAP2K2 | 8165 |
| apoptosis | MAP2K7 | 8171 |
| apoptosis | DIABLO | 8296 |
| apoptosis | BAD | 8540 |
| apoptosis | BAK1 | 8701 |
| apoptosis | BAX | 8750 |
| apoptosis | RAF1 | 8843 |
| apoptosis | BCL2 | 8920 |
| apoptosis | BCL2A1 | 8928 |
| apoptosis | BCL2L1 | 8937 |
| apoptosis | ROCK1 | 9045 |
| apoptosis | RPS6KA1 | 9117 |
| apoptosis | BID | 9260 |
| apoptosis | MAP2K4 | 9390 |
| apoptosis | SPTAN1 | 10209 |
| apoptosis | TNF | 10539 |
| apoptosis | TNFRSF1A | 10546 |
| apoptosis | TNFRSF1B | 10547 |
| apoptosis | TP53 | 10566 |
| apoptosis | CAPN1 | 12297 |
| apoptosis | CAPN2 | 12301 |
| apoptosis | CASP2 | 12393 |
| apoptosis | CASP3 | 12403 |
| apoptosis | CASP6 | 12446 |
| apoptosis | CASP7 | 12461 |
| apoptosis | CASP8 | 12479 |
| apoptosis | CASP9 | 12499 |
| apoptosis | CASP10 | 12519 |
| apoptosis | IKBKG | 12658 |
| apoptosis | MAP3K14 | 13097 |
| apoptosis | IKBKE | 13713 |
| apoptosis | CDC2 | 13897 |
| apoptosis | Casp12 | 120329 |
| breast cancer | CDK2 | 154 |
| breast cancer | CDK4 | 166 |
| breast cancer | NDRG1 | 347 |
| breast cancer | KNTC2 | 355 |
| breast cancer | PECI | 401 |
| breast cancer | CEACAM5 | 426 |
| breast cancer | SYNCRIP | 439 |
| breast cancer | PITRM1 | 472 |
| breast cancer | CENPA | 518 |
| breast cancer | IVNS1ABP | 559 |
| breast cancer | CENPF | 564 |
| breast cancer | RAI2 | 647 |
| breast cancer | CTSC | 655 |
| breast cancer | NMU | 736 |
| breast cancer | MAPRE2 | 828 |
| breast cancer | GCN1L1 | 830 |
| breast cancer | ESM1 | 916 |
| breast cancer | MGAT4A | 1105 |
| breast cancer | CIRBP | 1185 |
| breast cancer | CKS2 | 1204 |
| breast cancer | COL4A2 | 1334 |
| breast cancer | ADM | 1389 |
| breast cancer | CP | 1408 |
| breast cancer | CSF1 | 1506 |
| breast cancer | CSF3 | 1511 |
| breast cancer | CTNNB1 | 1595 |
| breast cancer | CTPS | 1605 |
| breast cancer | CTSB | 1613 |
| breast cancer | CTSD | 1614 |
| breast cancer | CTSE | 1617 |
| breast cancer | CTSL2 | 1628 |
| breast cancer | PAQR3 | 1646 |
| breast cancer | CYC1 | 1657 |
| breast cancer | CYP19A1 | 1713 |
| breast cancer | AP2B1 | 1760 |
| breast cancer | DCK | 1764 |
| breast cancer | ECT2 | 1968 |
| breast cancer | EGF | 2003 |
| breast cancer | EGFR | 2009 |
| breast cancer | EGR3 | 2013 |
| breast cancer | PTPLB | 2083 |
| breast cancer | ERBB2 | 2144 |
| breast cancer | ERBB3 | 2145 |
| breast cancer | ERBB4 | 2146 |
| breast cancer | AKT1 | 2150 |
| breast cancer | ESR1 | 2163 |
| breast cancer | ESR2 | 2166 |
| breast cancer | ALB | 2190 |
| breast cancer | EXT1 | 2192 |
| breast cancer | EZH2 | 2202 |
| breast cancer | FBP1 | 2261 |
| breast cancer | FGF3 | 2328 |
| breast cancer | FGF8 | 2334 |
| breast cancer | FLT1 | 2696 |
| breast cancer | FOS | 2959 |
| breast cancer | PRAME | 2962 |
| breast cancer | SEC14L2 | 2969 |
| breast cancer | ORC6L | 3009 |
| breast cancer | C20orf103 | 3103 |
| breast cancer | FUT8 | 3149 |
| breast cancer | PRKD2 | 3295 |
| breast cancer | C20orf28 | 3300 |
| breast cancer | MYRIP | 3331 |
| breast cancer | ASPM | 3333 |
| breast cancer | GAPDH | 3355 |
| breast cancer | KLK13 | 3429 |
| breast cancer | FBXO5 | 3523 |
| breast cancer | SACS | 3529 |
| breast cancer | GBE1 | 3547 |
| breast cancer | BBC3 | 3752 |
| breast cancer | PIB5PA | 3759 |
| breast cancer | EIF2C2 | 3775 |
| breast cancer | GNAZ | 3917 |
| breast cancer | GRB7 | 4099 |
| breast cancer | MRPL13 | 4143 |
| breast cancer | ATAD2 | 4155 |
| breast cancer | GSTM1 | 4247 |
| breast cancer | GSTM3 | 4249 |
| breast cancer | GPSM2 | 4320 |
| breast cancer | HMGB3 | 4541 |
| breast cancer | NR4A1 | 4553 |
| breast cancer | HRB | 4635 |
| breast cancer | BIRC5 | 4675 |
| breast cancer | IGF1 | 4811 |
| breast cancer | IGF2 | 4817 |
| breast cancer | IGFBP3 | 4824 |
| breast cancer | IGFBP5 | 4826 |
| breast cancer | ACADS | 4837 |
| breast cancer | INS | 4935 |
| breast cancer | AR | 4964 |
| breast cancer | ITGB3 | 4983 |
| breast cancer | JUN | 5012 |
| breast cancer | KRT18 | 5173 |
| breast cancer | KRT19 | 5188 |
| breast cancer | STMN1 | 5307 |
| breast cancer | MAD2L1 | 5485 |
| breast cancer | MATN3 | 5548 |
| breast cancer | MCM6 | 5572 |
| breast cancer | MKI67 | 5645 |
| breast cancer | MMP9 | 5670 |
| breast cancer | MMP11 | 5673 |
| breast cancer | ALDH6A1 | 5681 |
| breast cancer | ASNS | 5716 |
| breast cancer | MUC1 | 5846 |
| breast cancer | MX1 | 5860 |
| breast cancer | MYBL2 | 5867 |
| breast cancer | MYC | 5871 |
| breast cancer | ATM | 5968 |
| breast cancer | NMB | 6050 |
| breast cancer | NME1 | 6053 |
| breast cancer | OXCT1 | 6193 |
| breast cancer | PCSK6 | 6212 |
| breast cancer | SERPINE1 | 6232 |
| breast cancer | PCNA | 6403 |
| breast cancer | NUSAP1 | 6473 |
| breast cancer | PCTK1 | 6503 |
| breast cancer | UCHL5 | 6596 |
| breast cancer | IHPK2 | 6638 |
| breast cancer | EVL | 6650 |
| breast cancer | RAB6B | 6699 |
| breast cancer | PDPK1 | 6772 |
| breast cancer | PECAM1 | 6800 |
| breast cancer | PEX12 | 6834 |
| breast cancer | PFKP | 6854 |
| breast cancer | PGK1 | 6867 |
| breast cancer | PGR | 6872 |
| breast cancer | ABCB1 | 6873 |
| breast cancer | PLG | 6971 |
| breast cancer | EGLN1 | 7206 |
| breast cancer | VEGFA | 7422 |
| breast cancer | ARMC1 | 7588 |
| breast cancer | C20orf46 | 7749 |
| breast cancer | STK32B | 7775 |
| breast cancer | DKFZp762E1312 | 7777 |
| breast cancer | DEPDC1 | 7902 |
| breast cancer | ODZ3 | 7965 |
| breast cancer | PRKCA | 8025 |
| breast cancer | FGD6 | 8028 |
| breast cancer | PRKCB1 | 8032 |
| breast cancer | PRKCD | 8042 |
| breast cancer | PRKCE | 8050 |
| breast cancer | PRKCG | 8059 |
| breast cancer | PRKD1 | 8099 |
| breast cancer | PRKCZ | 8116 |
| breast cancer | MAPK3 | 8137 |
| breast cancer | PRL | 8223 |
| breast cancer | KIAA1217 | 8234 |
| breast cancer | SERF1A | 8297 |
| breast cancer | B2M | 8320 |
| breast cancer | MCCC1 | 8376 |
| breast cancer | CHPT1 | 8425 |
| breast cancer | PSMD2 | 8462 |
| breast cancer | HRASLS | 8484 |
| breast cancer | PSMD7 | 8498 |
| breast cancer | GPR126 | 8545 |
| breast cancer | PTEN | 8566 |
| breast cancer | BAG1 | 8570 |
| breast cancer | KIAA1324 | 8632 |
| breast cancer | SCUBE2 | 8691 |
| breast cancer | BAX | 8750 |
| breast cancer | RRAGD | 8802 |
| breast cancer | QDPR | 8809 |
| breast cancer | RAB27B | 8824 |
| breast cancer | RAD21 | 8834 |
| breast cancer | RAD51 | 8837 |
| breast cancer | RB1 | 8875 |
| breast cancer | PLEKHA1 | 8890 |
| breast cancer | RBP3 | 8911 |
| breast cancer | CCND1 | 8912 |
| breast cancer | BCL2 | 8920 |
| breast cancer | BCL2L1 | 8937 |
| breast cancer | RFC4 | 8942 |
| breast cancer | ACTB | 8958 |
| breast cancer | RPS4X | 9112 |
| breast cancer | RPS27A | 9145 |
| breast cancer | RRM2 | 9154 |
| breast cancer | SHBG | 9632 |
| breast cancer | SLC2A3 | 9883 |
| breast cancer | BMP6 | 10015 |
| breast cancer | SLC7A1 | 10020 |
| breast cancer | RASL11B | 10098 |
| breast cancer | BNIP3 | 10146 |
| breast cancer | SP1 | 10177 |
| breast cancer | ODZ1 | 10178 |
| breast cancer | SRC | 10215 |
| breast cancer | BRCA1 | 10220 |
| breast cancer | BRCA2 | 10245 |
| breast cancer | STK3 | 10280 |
| breast cancer | STX1A | 10289 |
| breast cancer | TBX3 | 10387 |
| breast cancer | BUB1 | 10418 |
| breast cancer | TFRC | 10459 |
| breast cancer | TGFA | 10461 |
| breast cancer | TGFB1 | 10462 |
| breast cancer | TGFB2 | 10464 |
| breast cancer | TGFB3 | 10465 |
| breast cancer | TK1 | 10501 |
| breast cancer | TNF | 10539 |
| breast cancer | TP53 | 10566 |
| breast cancer | TSG101 | 10623 |
| breast cancer | TYK2 | 10866 |
| breast cancer | VIM | 11129 |
| breast cancer | XRCC3 | 11183 |
| breast cancer | CA9 | 11256 |
| breast cancer | BTG2 | 11343 |
| breast cancer | LGP2 | 11458 |
| breast cancer | MLF1IP | 11624 |
| breast cancer | FBXO31 | 11710 |
| breast cancer | GSDMDC1 | 11711 |
| breast cancer | ST7 | 11735 |
| breast cancer | MLLT10 | 12031 |
| breast cancer | AKAP1 | 12222 |
| breast cancer | CDC42BPA | 12591 |
| breast cancer | PIR | 12697 |
| breast cancer | DEGS1 | 12719 |
| breast cancer | TMEFF1 | 12735 |
| breast cancer | ALDH4A1 | 12797 |
| breast cancer | FGF18 | 12926 |
| breast cancer | GMPS | 12941 |
| breast cancer | GGH | 12944 |
| breast cancer | WISP1 | 12948 |
| breast cancer | MTMR2 | 13000 |
| breast cancer | CCNB1 | 13010 |
| breast cancer | CCNE1 | 13055 |
| breast cancer | PRC1 | 13142 |
| breast cancer | CCNB2 | 13228 |
| breast cancer | CCNE2 | 13229 |
| breast cancer | TRIP13 | 13420 |
| breast cancer | KIF3B | 13474 |
| breast cancer | ABCG2 | 13531 |
| breast cancer | CD34 | 13567 |
| breast cancer | BAG3 | 13621 |
| breast cancer | CD68 | 13752 |
| breast cancer | DLG7 | 13859 |
| breast cancer | PTDSS1 | 13863 |
| breast cancer | MELK | 13901 |
| breast cancer | KIF14 | 13993 |
| breast cancer | CDC25B | 14004 |
| breast cancer | CDH1 | 14047 |
| breast cancer | KIF21A | 55605 |
| breast cancer | Spc25 | 57405 |
| breast cancer | MIB1 | 57534 |
| breast cancer | MS4A7 | 58475 |
| breast cancer | KIAA1683 | 80726 |
| breast cancer | MUC19 | 283463 |
| cell cycle | RAD50 | 100 |
| cell cycle | CDK2 | 154 |
| cell cycle | CDK4 | 166 |
| cell cycle | CDK6 | 187 |
| cell cycle | CDK7 | 198 |
| cell cycle | CDK8 | 218 |
| cell cycle | CDKN1A | 237 |
| cell cycle | CDKN1B | 247 |
| cell cycle | CDKN1C | 256 |
| cell cycle | CDKN2A | 266 |
| cell cycle | CDKN2B | 277 |
| cell cycle | CDKN2C | 284 |
| cell cycle | CDKN2D | 293 |
| cell cycle | CDKN3 | 303 |
| cell cycle | CHEK1 | 935 |
| cell cycle | CDC37 | 960 |
| cell cycle | CHEK2 | 1019 |
| cell cycle | CKS1B | 1202 |
| cell cycle | CKS2 | 1204 |
| cell cycle | GADD45A | 1779 |
| cell cycle | DDX11 | 1793 |
| cell cycle | DNM2 | 1881 |
| cell cycle | E2F1 | 1953 |
| cell cycle | E2F2 | 1955 |
| cell cycle | E2F3 | 1956 |
| cell cycle | E2F4 | 1957 |
| cell cycle | E2F5 | 1958 |
| cell cycle | E2F6 | 1959 |
| cell cycle | ABL1 | 3132 |
| cell cycle | GAPDH | 3355 |
| cell cycle | RGC32 | 4136 |
| cell cycle | GTF2H1 | 4264 |
| cell cycle | ANAPC2 | 4309 |
| cell cycle | BIRC5 | 4675 |
| cell cycle | HUS1 | 4705 |
| cell cycle | KPNA2 | 5140 |
| cell cycle | MAD2L1 | 5485 |
| cell cycle | MCM2 | 5568 |
| cell cycle | MCM3 | 5569 |
| cell cycle | MCM4 | 5570 |
| cell cycle | MCM5 | 5571 |
| cell cycle | MCM6 | 5572 |
| cell cycle | MCM7 | 5573 |
| cell cycle | MKI67 | 5645 |
| cell cycle | MNAT1 | 5684 |
| cell cycle | MRE11A | 5706 |
| cell cycle | RBL1 | 5933 |
| cell cycle | ATM | 5968 |
| cell cycle | PCNA | 6403 |
| cell cycle | HERC5 | 6464 |
| cell cycle | ANAPC5 | 6629 |
| cell cycle | GTSE1 | 6676 |
| cell cycle | CDK5RAP1 | 6752 |
| cell cycle | ATR | 7152 |
| cell cycle | BCCIP | 8302 |
| cell cycle | B2M | 8320 |
| cell cycle | BAX | 8750 |
| cell cycle | RAD1 | 8751 |
| cell cycle | RAD9A | 8832 |
| cell cycle | RAD17 | 8833 |
| cell cycle | RAD51 | 8837 |
| cell cycle | RB1 | 8875 |
| cell cycle | RBBP8 | 8887 |
| cell cycle | RBL2 | 8892 |
| cell cycle | CCND1 | 8912 |
| cell cycle | BCL2 | 8920 |
| cell cycle | ACTB | 8958 |
| cell cycle | RPA3 | 9057 |
| cell cycle | RPS27A | 9145 |
| cell cycle | SKP2 | 9824 |
| cell cycle | BRCA1 | 10220 |
| cell cycle | BRCA2 | 10245 |
| cell cycle | TFDP1 | 10451 |
| cell cycle | TFDP2 | 10452 |
| cell cycle | MAD2L2 | 10459 |
| cell cycle | TP53 | 10566 |
| cell cycle | UBE1 | 10997 |
| cell cycle | SUMO1 | 11062 |
| cell cycle | CDK5RAP3 | 12030 |
| cell cycle | CUL5 | 12073 |
| cell cycle | CDC7 | 12335 |
| cell cycle | CDC45L | 12336 |
| cell cycle | CUL4A | 12546 |
| cell cycle | CUL3 | 12548 |
| cell cycle | CUL2 | 12550 |
| cell cycle | CUL1 | 12551 |
| cell cycle | CDK5R1 | 12958 |
| cell cycle | CDC16 | 12984 |
| cell cycle | CCNA2 | 13003 |
| cell cycle | CCNA1 | 13004 |
| cell cycle | CCNB1 | 13010 |
| cell cycle | CCNC | 13018 |
| cell cycle | CCND2 | 13035 |
| cell cycle | CDK5R2 | 13037 |
| cell cycle | CCND3 | 13041 |
| cell cycle | CCNE1 | 13055 |
| cell cycle | CCNF | 13066 |
| cell cycle | CCNG1 | 13082 |
| cell cycle | CCNG2 | 13085 |
| cell cycle | CCNH | 13096 |
| cell cycle | CCNT1 | 13122 |
| cell cycle | CCNT2 | 13136 |
| cell cycle | PKMYT1 | 13175 |
| cell cycle | CCNB2 | 13228 |
| cell cycle | CCNE2 | 13229 |
| cell cycle | KNTC1 | 13808 |
| cell cycle | CDC2 | 13897 |
| cell cycle | CDC6 | 13964 |
| cell cycle | CDC20 | 13975 |
| cell cycle | CDC25A | 13995 |
| cell cycle | CDC25C | 14014 |
| cell cycle | CDC34 | 14029 |
| cell cycle | ANAPC4 | 29945 |
| cell cycle | SERTAD1 | 29950 |
| cell surface signaling | CDH5 | 31 |
| cell surface signaling | ADAM8 | 89 |
| cell surface signaling | CD96 | 203 |
| cell surface signaling | PROCR | 484 |
| cell surface signaling | CD160 | 947 |
| cell surface signaling | CHIT1 | 998 |
| cell surface signaling | CMA1 | 1264 |
| cell surface signaling | COL1A1 | 1326 |
| cell surface signaling | COL1A2 | 1328 |
| cell surface signaling | COL3A1 | 1331 |
| cell surface signaling | CR2 | 1433 |
| cell surface signaling | CSF1R | 1507 |
| cell surface signaling | CTLA4 | 1588 |
| cell surface signaling | ACE | 1767 |
| cell surface signaling | DPP4 | 1897 |
| cell surface signaling | ENG | 2097 |
| cell surface signaling | ALCAM | 2199 |
| cell surface signaling | FABP4 | 2220 |
| cell surface signaling | FCER1A | 2263 |
| cell surface signaling | FCER2 | 2269 |
| cell surface signaling | FCGR1A | 2271 |
| cell surface signaling | FCGR3B | 2289 |
| cell surface signaling | GAPDH | 3355 |
| cell surface signaling | ITGA1 | 3672 |
| cell surface signaling | CD209 | 4480 |
| cell surface signaling | ICAM2 | 4715 |
| cell surface signaling | FAS | 4861 |
| cell surface signaling | IL2RA | 4870 |
| cell surface signaling | IL12RB1 | 4900 |
| cell surface signaling | ITGA2 | 4967 |
| cell surface signaling | ITGA3 | 4969 |
| cell surface signaling | KLRB1 | 5126 |
| cell surface signaling | KLRC1 | 5127 |
| cell surface signaling | KLRD1 | 5130 |
| cell surface signaling | KRT4 | 5152 |
| cell surface signaling | KRT5 | 5153 |
| cell surface signaling | KRT8 | 5157 |
| cell surface signaling | KRT10 | 5159 |
| cell surface signaling | KRT13 | 5161 |
| cell surface signaling | KRT17 | 5169 |
| cell surface signaling | KRT18 | 5173 |
| cell surface signaling | KRT19 | 5188 |
| cell surface signaling | LAG3 | 5253 |
| cell surface signaling | TACSTD1 | 5475 |
| cell surface signaling | CD22 | 5497 |
| cell surface signaling | MCAM | 5563 |
| cell surface signaling | MUC1 | 5846 |
| cell surface signaling | MYH9 | 5886 |
| cell surface signaling | MYH10 | 5887 |
| cell surface signaling | MYH11 | 5888 |
| cell surface signaling | NCAM1 | 5934 |
| cell surface signaling | NOS3 | 6064 |
| cell surface signaling | NT5E | 6113 |
| cell surface signaling | CD244 | 6798 |
| cell surface signaling | PECAM1 | 6800 |
| cell surface signaling | B2M | 8320 |
| cell surface signaling | RETN | 8324 |
| cell surface signaling | ACTA2 | 8850 |
| cell surface signaling | ACTB | 8958 |
| cell surface signaling | RPS27A | 9145 |
| cell surface signaling | S100A8 | 9177 |
| cell surface signaling | SELE | 9331 |
| cell surface signaling | SELP | 9333 |
| cell surface signaling | ST6GAL1 | 9732 |
| cell surface signaling | FSCN1 | 10131 |
| cell surface signaling | TEK | 10436 |
| cell surface signaling | THBD | 10478 |
| cell surface signaling | TPSAB1 | 10586 |
| cell surface signaling | TNFRSF4 | 10830 |
| cell surface signaling | VCAM1 | 11115 |
| cell surface signaling | VWF | 11143 |
| cell surface signaling | IL1R2 | 11352 |
| cell surface signaling | CD1A | 13176 |
| cell surface signaling | CD1C | 13197 |
| cell surface signaling | CD1D | 13207 |
| cell surface signaling | CD2 | 13238 |
| cell surface signaling | CD3D | 13251 |
| cell surface signaling | CD3G | 13272 |
| cell surface signaling | CD4 | 13304 |
| cell surface signaling | CD5 | 13316 |
| cell surface signaling | CD6 | 13339 |
| cell surface signaling | CD7 | 13349 |
| cell surface signaling | CD8A | 13360 |
| cell surface signaling | CD14 | 13398 |
| cell surface signaling | CD19 | 13405 |
| cell surface signaling | CD83 | 13407 |
| cell surface signaling | MS4A1 | 13409 |
| cell surface signaling | CD163 | 13434 |
| cell surface signaling | CD24 | 13440 |
| cell surface signaling | ADIPOQ | 13473 |
| cell surface signaling | CD28 | 13498 |
| cell surface signaling | CD80 | 13510 |
| cell surface signaling | CD86 | 13522 |
| cell surface signaling | TNFRSF8 | 13532 |
| cell surface signaling | CD33 | 13548 |
| cell surface signaling | CD34 | 13567 |
| cell surface signaling | CHST10 | 13579 |
| cell surface signaling | CD37 | 13599 |
| cell surface signaling | CD38 | 13609 |
| cell surface signaling | CD40 | 13661 |
| cell surface signaling | CD40LG | 13672 |
| cell surface signaling | CD63 | 13741 |
| cell surface signaling | CD68 | 13752 |
| cell surface signaling | CD69 | 13762 |
| cell surface signaling | CD72 | 13783 |
| cell surface signaling | CD74 | 13792 |
| cell surface signaling | CD79A | 13802 |
| cell surface signaling | CD79B | 13813 |
| cell surface signaling | MYOCD | 93649 |
| chemokines | CFL1 | 629 |
| chemokines | CCR3 | 1271 |
| chemokines | CCR5 | 1274 |
| chemokines | MAPK14 | 1504 |
| chemokines | PLCG1 | 1630 |
| chemokines | PTK2B | 2236 |
| chemokines | PLCB1 | 2723 |
| chemokines | FOS | 2959 |
| chemokines | NOX1 | 3709 |
| chemokines | GNAI1 | 3907 |
| chemokines | GNAI2 | 3908 |
| chemokines | GNAQ | 3912 |
| chemokines | HRAS | 4634 |
| chemokines | JUN | 5012 |
| chemokines | KRAS | 5147 |
| chemokines | RHOA | 5166 |
| chemokines | LIMK1 | 5354 |
| chemokines | MYL2 | 5891 |
| chemokines | PPP1R12A | 5911 |
| chemokines | PPP1R12B | 5913 |
| chemokines | NRAS | 6102 |
| chemokines | PIK3C2G | 6909 |
| chemokines | PLCB2 | 6949 |
| chemokines | PLCB3 | 6950 |
| chemokines | PLCB4 | 6951 |
| chemokines | PLCG2 | 6964 |
| chemokines | PRKCA | 8025 |
| chemokines | PRKCB1 | 8032 |
| chemokines | MAPK1 | 8136 |
| chemokines | MAPK3 | 8137 |
| chemokines | MAPK8 | 8152 |
| chemokines | MAP2K1 | 8164 |
| chemokines | MAP2K2 | 8165 |
| chemokines | PTK2 | 8616 |
| chemokines | RAF1 | 8843 |
| chemokines | CCL2 | 9238 |
| chemokines | CCL4 | 9242 |
| chemokines | CCL5 | 9243 |
| chemokines | CCL7 | 9244 |
| chemokines | CCL11 | 9246 |
| chemokines | CCL13 | 9247 |
| chemokines | CCL24 | 9259 |
| chemokines | CXCL12 | 9274 |
| chemokines | SRC | 10215 |
| chemokines | CXCR4 | 11354 |
| chemokines | CALM1 | 11933 |
| chemokines | CALM2 | 12070 |
| chemokines | CALM3 | 12111 |
| chemokines | CAMK4 | 12157 |
| chemokines | CAMK2A | 12166 |
| chemokines | CAMK2B | 12198 |
| chemokines | CAMK2D | 12232 |
| chemokines | CAMK2G | 12243 |
| chemokines | CAMK1 | 12681 |
| chemokines | ROCK2 | 13572 |
| DNA damage | RAD50 | 100 |
| DNA damage | CDK7 | 198 |
| DNA damage | UNG2 | 283 |
| DNA damage | CIB1 | 458 |
| DNA damage | GADD45G | 768 |
| DNA damage | CHEK1 | 935 |
| DNA damage | DMC1 | 964 |
| DNA damage | CHEK2 | 1019 |
| DNA damage | TREX2 | 1030 |
| DNA damage | TREX1 | 1078 |
| DNA damage | PNKP | 1085 |
| DNA damage | CIDEA | 1176 |
| DNA damage | ERCC8 | 1199 |
| DNA damage | CRY1 | 1477 |
| DNA damage | CRY2 | 1479 |
| DNA damage | DDB1 | 1773 |
| DNA damage | DDB2 | 1774 |
| DNA damage | GADD45A | 1779 |
| DNA damage | DDIT3 | 1780 |
| DNA damage | ERCC1 | 2147 |
| DNA damage | ERCC2 | 2148 |
| DNA damage | ERCC3 | 2152 |
| DNA damage | ERCC4 | 2153 |
| DNA damage | ERCC5 | 2154 |
| DNA damage | FANCG | 2239 |
| DNA damage | FEN1 | 2318 |
| DNA damage | PPP1R15A | 3047 |
| DNA damage | ABL1 | 3132 |
| DNA damage | GAPDH | 3355 |
| DNA damage | ANKRD17 | 3418 |
| DNA damage | MLH3 | 3705 |
| DNA damage | CIDEB | 3770 |
| DNA damage | SESN1 | 3805 |
| DNA damage | APEX2 | 3836 |
| DNA damage | GML | 3901 |
| DNA damage | MSH6 | 4256 |
| DNA damage | GTF2H1 | 4264 |
| DNA damage | GTF2H2 | 4265 |
| DNA damage | GTF2H3 | 4266 |
| DNA damage | APEX1 | 4644 |
| DNA damage | HUS1 | 4705 |
| DNA damage | IGHMBP2 | 4844 |
| DNA damage | INPPL1 | 4940 |
| DNA damage | LIG1 | 5348 |
| DNA damage | LIG3 | 5350 |
| DNA damage | LIG4 | 5351 |
| DNA damage | MLH1 | 5649 |
| DNA damage | MNAT1 | 5684 |
| DNA damage | MPG | 5695 |
| DNA damage | MRE11A | 5706 |
| DNA damage | PMS2 | 5765 |
| DNA damage | MSH2 | 5803 |
| DNA damage | MSH3 | 5804 |
| DNA damage | MSH4 | 5805 |
| DNA damage | MSH5 | 5806 |
| DNA damage | NUDT1 | 5834 |
| DNA damage | MUTYH | 5857 |
| DNA damage | ATM | 5968 |
| DNA damage | NTHL1 | 6116 |
| DNA damage | OGG1 | 6153 |
| DNA damage | PCNA | 6403 |
| DNA damage | GTSE1 | 6676 |
| DNA damage | ZAK | 6816 |
| DNA damage | PMS1 | 7011 |
| DNA damage | PMS2L4 | 7015 |
| DNA damage | PMS2L3 | 7027 |
| DNA damage | ATR | 7152 |
| DNA damage | ATRX | 7212 |
| DNA damage | PRKDC | 8124 |
| DNA damage | MAP2K6 | 8170 |
| DNA damage | B2M | 8320 |
| DNA damage | PCBP4 | 8458 |
| DNA damage | RAD1 | 8751 |
| DNA damage | RAD9A | 8832 |
| DNA damage | RAD17 | 8833 |
| DNA damage | RAD21 | 8834 |
| DNA damage | RAD23A | 8835 |
| DNA damage | RAD23B | 8836 |
| DNA damage | RAD51 | 8837 |
| DNA damage | RAD51C | 8838 |
| DNA damage | RAD51L1 | 8839 |
| DNA damage | RAD51L3 | 8841 |
| DNA damage | RAD52 | 8842 |
| DNA damage | RBBP8 | 8887 |
| DNA damage | ACTB | 8958 |
| DNA damage | RPA1 | 9055 |
| DNA damage | RPA3 | 9057 |
| DNA damage | RPS27A | 9145 |
| DNA damage | MAPK12 | 9196 |
| DNA damage | SEMA4A | 9418 |
| DNA damage | BRCA1 | 10220 |
| DNA damage | BRCA2 | 10245 |
| DNA damage | TP53 | 10566 |
| DNA damage | TP73 | 10570 |
| DNA damage | SUMO1 | 11062 |
| DNA damage | UNG | 11087 |
| DNA damage | XPA | 11176 |
| DNA damage | XPC | 11177 |
| DNA damage | XRCC1 | 11181 |
| DNA damage | XRCC2 | 11182 |
| DNA damage | XRCC3 | 11183 |
| DNA damage | XRCC4 | 11184 |
| DNA damage | XRCC5 | 11187 |
| DNA damage | BTG2 | 11343 |
| DNA damage | RAD54L | 12530 |
| DNA damage | MBD4 | 13027 |
| DNA damage | CCNH | 13096 |
| DNA damage | EXO1 | 13257 |
| DNA damage | N4BP2 | 55728 |
| DNA damage | RAD18 | 56852 |
| DNA damage | IHPK3 | 117283 |
| DNA repair | PARP3 | 34 |
| DNA repair | RAD50 | 100 |
| DNA repair | CDK7 | 198 |
| DNA repair | UNG2 | 283 |
| DNA repair | CIB1 | 458 |
| DNA repair | CETN2 | 613 |
| DNA repair | POLD3 | 626 |
| DNA repair | DMC1 | 964 |
| DNA repair | TREX1 | 1078 |
| DNA repair | PNKP | 1085 |
| DNA repair | ERCC8 | 1199 |
| DNA repair | PARP1 | 1496 |
| DNA repair | DDB1 | 1773 |
| DNA repair | DDB2 | 1774 |
| DNA repair | DKC1 | 1844 |
| DNA repair | ERCC1 | 2147 |
| DNA repair | ERCC2 | 2148 |
| DNA repair | ERCC3 | 2152 |
| DNA repair | ERCC4 | 2153 |
| DNA repair | ERCC5 | 2154 |
| DNA repair | ERCC6 | 2155 |
| DNA repair | FEN1 | 2318 |
| DNA repair | SMUG1 | 2998 |
| DNA repair | RAD54B | 3234 |
| DNA repair | GAPDH | 3355 |
| DNA repair | ANKRD17 | 3418 |
| DNA repair | TINF2 | 3528 |
| DNA repair | MLH3 | 3705 |
| DNA repair | APEX2 | 3836 |
| DNA repair | POLL | 3865 |
| DNA repair | MSH6 | 4256 |
| DNA repair | GTF2H1 | 4264 |
| DNA repair | GTF2H2 | 4265 |
| DNA repair | GTF2H3 | 4266 |
| DNA repair | GTF2H4 | 4267 |
| DNA repair | APEX1 | 4644 |
| DNA repair | LIG1 | 5348 |
| DNA repair | LIG3 | 5350 |
| DNA repair | LIG4 | 5351 |
| DNA repair | MGMT | 5629 |
| DNA repair | ATXN3 | 5644 |
| DNA repair | MLH1 | 5649 |
| DNA repair | MNAT1 | 5684 |
| DNA repair | MPG | 5695 |
| DNA repair | MRE11A | 5706 |
| DNA repair | PMS2 | 5765 |
| DNA repair | MSH2 | 5803 |
| DNA repair | MSH3 | 5804 |
| DNA repair | MSH4 | 5805 |
| DNA repair | MSH5 | 5806 |
| DNA repair | MUTYH | 5857 |
| DNA repair | ATM | 5968 |
| DNA repair | NTHL1 | 6116 |
| DNA repair | OGG1 | 6153 |
| DNA repair | PMS1 | 7011 |
| DNA repair | PMS2L1 | 7012 |
| DNA repair | PMS2L3 | 7027 |
| DNA repair | TERF2IP | 7102 |
| DNA repair | ATR | 7152 |
| DNA repair | PRKDC | 8124 |
| DNA repair | B2M | 8320 |
| DNA repair | XAB2 | 8398 |
| DNA repair | RAD21 | 8834 |
| DNA repair | RAD23A | 8835 |
| DNA repair | RAD23B | 8836 |
| DNA repair | RAD51 | 8837 |
| DNA repair | RAD51C | 8838 |
| DNA repair | RAD51L1 | 8839 |
| DNA repair | RAD51L3 | 8841 |
| DNA repair | RAD52 | 8842 |
| DNA repair | RFC1 | 8939 |
| DNA repair | ACTB | 8958 |
| DNA repair | RPA1 | 9055 |
| DNA repair | RPA3 | 9057 |
| DNA repair | RPS27A | 9145 |
| DNA repair | MMS19L | 9411 |
| DNA repair | TDG | 9587 |
| DNA repair | BRCA1 | 10220 |
| DNA repair | BRCA2 | 10245 |
| DNA repair | TEP1 | 10437 |
| DNA repair | TERF1 | 10438 |
| DNA repair | TERF2 | 10439 |
| DNA repair | TERT | 10440 |
| DNA repair | TOP3A | 10565 |
| DNA repair | TP73 | 10570 |
| DNA repair | UBE2A | 11011 |
| DNA repair | UBE2B | 11020 |
| DNA repair | UBE2I | 11054 |
| DNA repair | UBE2N | 11057 |
| DNA repair | UBE2V2 | 11059 |
| DNA repair | UNG | 11087 |
| DNA repair | XPA | 11176 |
| DNA repair | XPC | 11177 |
| DNA repair | XRCC1 | 11181 |
| DNA repair | XRCC2 | 11182 |
| DNA repair | XRCC3 | 11183 |
| DNA repair | XRCC4 | 11184 |
| DNA repair | XRCC5 | 11187 |
| DNA repair | NEIL1 | 11607 |
| DNA repair | TNKS2 | 12063 |
| DNA repair | BRIP1 | 12460 |
| DNA repair | RAD54L | 12530 |
| DNA repair | TNKS | 12796 |
| DNA repair | MBD4 | 13027 |
| DNA repair | TOP3B | 13036 |
| DNA repair | CCNH | 13096 |
| DNA repair | EXO1 | 13257 |
| DNA repair | SLK | 13821 |
| DNA repair | N4BP2 | 55728 |
| DNA repair | RAD18 | 56852 |
| DNA repair | TNKS1BP1 | 85456 |
| DNA repair | NEIL2 | 252969 |
| EGF | CDKN1A | 237 |
| EGF | CNTF | 1320 |
| EGF | CPT1A | 1427 |
| EGF | CREB1 | 1440 |
| EGF | CRP | 1461 |
| EGF | CSNK2A1 | 1531 |
| EGF | CSNK2A2 | 1534 |
| EGF | CSNK2B | 1536 |
| EGF | PLCG1 | 1630 |
| EGF | DNMT1 | 1882 |
| EGF | EGF | 2003 |
| EGF | EGFR | 2009 |
| EGF | EGR1 | 2010 |
| EGF | A2M | 2053 |
| EGF | ELK1 | 2059 |
| EGF | ELK1 | 2059 |
| EGF | ENO2 | 2101 |
| EGF | F3 | 2208 |
| EGF | FOS | 2959 |
| EGF | FOSB | 2968 |
| EGF | GATA4 | 3517 |
| EGF | GFAP | 3641 |
| EGF | GRB2 | 4098 |
| EGF | GZMA | 4398 |
| EGF | HGF | 4475 |
| EGF | NR4A1 | 4553 |
| EGF | HP | 4623 |
| EGF | HRAS | 4634 |
| EGF | BIRC5 | 4675 |
| EGF | ICAM1 | 4714 |
| EGF | IGFBP1 | 4822 |
| EGF | APP | 4845 |
| EGF | KLK3 | 4857 |
| EGF | IL1RN | 4868 |
| EGF | IL6 | 4880 |
| EGF | ITGAM | 4977 |
| EGF | ITGB2 | 4981 |
| EGF | ITPR1 | 5001 |
| EGF | ITPR2 | 5002 |
| EGF | ITPR3 | 5003 |
| EGF | JAK1 | 5007 |
| EGF | JUN | 5012 |
| EGF | JUNB | 5013 |
| EGF | KRT16 | 5165 |
| EGF | LY6H | 5463 |
| EGF | MAF | 5495 |
| EGF | MAP3K1 | 5600 |
| EGF | MMP1 | 5665 |
| EGF | MMP2 | 5666 |
| EGF | MMP13 | 5675 |
| EGF | MMP14 | 5676 |
| EGF | MYC | 5871 |
| EGF | MYOD1 | 5909 |
| EGF | NPPA | 6086 |
| EGF | PIK3CA | 6912 |
| EGF | PIK3CB | 6913 |
| EGF | PIK3CD | 6915 |
| EGF | PIK3R1 | 6917 |
| EGF | PIK3R1 | 6917 |
| EGF | PIK3R2 | 6918 |
| EGF | VEGFA | 7422 |
| EGF | PRKCA | 8025 |
| EGF | MAPK1 | 8136 |
| EGF | MAPK3 | 8137 |
| EGF | MAPK8 | 8152 |
| EGF | MAP2K1 | 8164 |
| EGF | MAP2K7 | 8171 |
| EGF | RAF1 | 8843 |
| EGF | RASA1 | 8872 |
| EGF | CCND1 | 8912 |
| EGF | BCL2 | 8920 |
| EGF | REG1A | 8925 |
| EGF | BCL2L1 | 8937 |
| EGF | RPS6KB1 | 9120 |
| EGF | SAA2 | 9186 |
| EGF | CCL2 | 9238 |
| EGF | CCL11 | 9246 |
| EGF | MAP2K4 | 9390 |
| EGF | SHC1 | 9640 |
| EGF | SOS1 | 10164 |
| EGF | SOS2 | 10165 |
| EGF | SRF | 10223 |
| EGF | STAT1 | 10264 |
| EGF | STAT3 | 10266 |
| EGF | TGFB1 | 10462 |
| EGF | TH | 10477 |
| EGF | VIP | 11130 |
| EGF | WEE1 | 11152 |
| EGF | CA2 | 11236 |
| EGF | FOSL1 | 12072 |
| EGF | CAMK2A | 12166 |
| EGF | PIK3R3 | 12644 |
| EGF | SOCS1 | 12792 |
| EGF | CCNB1 | 13010 |
| EGF | SOCS3 | 13099 |
| EGF | CD9 | 13388 |
| EGF | IER2 | 13674 |
| EGF | CDC2 | 13897 |
| EGF | KCNH8 | 131096 |
| FGF | CST8 | 42 |
| FGF | CDKN1A | 237 |
| FGF | CEBPB | 452 |
| FGF | CFTR | 690 |
| FGF | FRS2 | 704 |
| FGF | RUVBL2 | 722 |
| FGF | PPARGC1A | 748 |
| FGF | CNTF | 1320 |
| FGF | CREB1 | 1440 |
| FGF | ATF2 | 1441 |
| FGF | CREM | 1445 |
| FGF | CRH | 1447 |
| FGF | CRK | 1456 |
| FGF | CRP | 1461 |
| FGF | MAPK14 | 1504 |
| FGF | AANAT | 1598 |
| FGF | PLCG1 | 1630 |
| FGF | CYP11A1 | 1705 |
| FGF | CYP19A1 | 1713 |
| FGF | DCN | 1765 |
| FGF | EGR1 | 2010 |
| FGF | A2M | 2053 |
| FGF | ELK1 | 2059 |
| FGF | ENO2 | 2101 |
| FGF | FGF10 | 2255 |
| FGF | FGF11 | 2256 |
| FGF | FGF1 | 2326 |
| FGF | FGF2 | 2327 |
| FGF | FGF3 | 2328 |
| FGF | FGF4 | 2329 |
| FGF | FGF5 | 2331 |
| FGF | FGF6 | 2332 |
| FGF | FGF7 | 2333 |
| FGF | FGF8 | 2334 |
| FGF | FGF9 | 2335 |
| FGF | FGF12 | 2336 |
| FGF | FGF13 | 2337 |
| FGF | FGF14 | 2338 |
| FGF | FGFR1 | 2340 |
| FGF | FGFR3 | 2341 |
| FGF | FGFR2 | 2343 |
| FGF | FGFR4 | 2344 |
| FGF | FOS | 2959 |
| FGF | FOSL2 | 2978 |
| FGF | GAB1 | 3178 |
| FGF | FGF20 | 3533 |
| FGF | FGF21 | 3541 |
| FGF | GFAP | 3641 |
| FGF | FGF22 | 3693 |
| FGF | ANG | 3971 |
| FGF | MKNK2 | 4080 |
| FGF | GRB2 | 4098 |
| FGF | GZMA | 4398 |
| FGF | ANXA1 | 4410 |
| FGF | HGF | 4475 |
| FGF | HP | 4623 |
| FGF | HRAS | 4634 |
| FGF | BIRC5 | 4675 |
| FGF | ICAM1 | 4714 |
| FGF | APP | 4845 |
| FGF | KLK3 | 4857 |
| FGF | IL2 | 4869 |
| FGF | IL6 | 4880 |
| FGF | INS | 4935 |
| FGF | ITGAM | 4977 |
| FGF | ITGB2 | 4981 |
| FGF | ITPR1 | 5001 |
| FGF | JUN | 5012 |
| FGF | LDLR | 5326 |
| FGF | LY6H | 5463 |
| FGF | MAF | 5495 |
| FGF | MAP3K1 | 5600 |
| FGF | MAP3K5 | 5603 |
| FGF | MMP1 | 5665 |
| FGF | MMP2 | 5666 |
| FGF | MYC | 5871 |
| FGF | ATF3 | 5920 |
| FGF | NFKB1 | 6020 |
| FGF | NGFB | 6033 |
| FGF | SERPINE1 | 6232 |
| FGF | PCK1 | 6353 |
| FGF | PCNA | 6403 |
| FGF | PCSK1 | 6483 |
| FGF | PDGFRA | 6698 |
| FGF | PIK3R1 | 6917 |
| FGF | PPARG | 7238 |
| FGF | VEGFA | 7422 |
| FGF | PRKCA | 8025 |
| FGF | MAPK1 | 8136 |
| FGF | MAPK3 | 8137 |
| FGF | MAPK8 | 8152 |
| FGF | MAP2K1 | 8164 |
| FGF | MAP2K3 | 8166 |
| FGF | MAP2K6 | 8170 |
| FGF | PTPN11 | 8705 |
| FGF | RAC1 | 8829 |
| FGF | RAF1 | 8843 |
| FGF | RB1 | 8875 |
| FGF | CCND1 | 8912 |
| FGF | BCL2 | 8920 |
| FGF | REG1A | 8925 |
| FGF | BCL2L1 | 8937 |
| FGF | SAA2 | 9186 |
| FGF | CCL11 | 9246 |
| FGF | FGF19 | 9965 |
| FGF | SOD2 | 10157 |
| FGF | SOS1 | 10164 |
| FGF | SST | 10246 |
| FGF | STAT3 | 10266 |
| FGF | TH | 10477 |
| FGF | NR2C2 | 10591 |
| FGF | VIP | 11130 |
| FGF | FGF23 | 12083 |
| FGF | MKNK1 | 12727 |
| FGF | SOCS1 | 12792 |
| FGF | RIPK2 | 12887 |
| FGF | FGF18 | 12926 |
| FGF | FGF17 | 12931 |
| FGF | FGF16 | 12932 |
| FGF | CCK | 12956 |
| FGF | CCNB1 | 13010 |
| FGF | SOCS3 | 13099 |
| FGF | MAPKAPK2 | 13373 |
| FGF | CD9 | 13388 |
| FGF | IER2 | 13674 |
| FGF | CDC2 | 13897 |
| G1_S | HDAC5 | 17 |
| G1_S | CDK2 | 154 |
| G1_S | CDK4 | 166 |
| G1_S | CDK6 | 187 |
| G1_S | CDKN1A | 237 |
| G1_S | CDKN1B | 247 |
| G1_S | CDKN2A | 266 |
| G1_S | CDKN2B | 277 |
| G1_S | DHFR | 1832 |
| G1_S | E2F1 | 1953 |
| G1_S | E2F2 | 1955 |
| G1_S | E2F3 | 1956 |
| G1_S | E2F4 | 1957 |
| G1_S | E2F5 | 1958 |
| G1_S | E2F6 | 1959 |
| G1_S | ABL1 | 3132 |
| G1_S | GSK3B | 4239 |
| G1_S | HDAC1 | 4454 |
| G1_S | HDAC2 | 4455 |
| G1_S | SMAD3 | 5488 |
| G1_S | SMAD4 | 5489 |
| G1_S | MYC | 5871 |
| G1_S | RBL1 | 5933 |
| G1_S | RBL1 | 5933 |
| G1_S | ATM | 5968 |
| G1_S | PCNA | 6403 |
| G1_S | ATR | 7152 |
| G1_S | RANGAP1 | 8856 |
| G1_S | RB1 | 8875 |
| G1_S | RBL2 | 8892 |
| G1_S | CCND1 | 8912 |
| G1_S | SKP1A | 9814 |
| G1_S | SUV39H1 | 10312 |
| G1_S | TFDP1 | 10451 |
| G1_S | TGFB1 | 10462 |
| G1_S | TGFB2 | 10464 |
| G1_S | TGFB3 | 10465 |
| G1_S | TK1 | 10501 |
| G1_S | TP53 | 10566 |
| G1_S | CUL1 | 12551 |
| G1_S | HDAC3 | 12949 |
| G1_S | CCND2 | 13035 |
| G1_S | BTRC | 13040 |
| G1_S | CCND3 | 13041 |
| G1_S | CCNE1 | 13055 |
| G1_S | CCNE2 | 13229 |
| G1_S | HDAC4 | 13831 |
| G1_S | CDC2 | 13897 |
| G1_S | CDC25A | 13995 |
| G2_M | CDK7 | 198 |
| G2_M | CDKN1A | 237 |
| G2_M | CDKN2A | 266 |
| G2_M | CHEK1 | 935 |
| G2_M | CHEK2 | 1019 |
| G2_M | GADD45A | 1779 |
| G2_M | EP300 | 2110 |
| G2_M | SFN | 3947 |
| G2_M | MDM2 | 5583 |
| G2_M | MYT1 | 5914 |
| G2_M | ATM | 5968 |
| G2_M | PLK1 | 6979 |
| G2_M | ATR | 7152 |
| G2_M | PRKDC | 8124 |
| G2_M | RPRM | 8276 |
| G2_M | RPS6KA1 | 9117 |
| G2_M | SKP1A | 9814 |
| G2_M | BRCA1 | 10220 |
| G2_M | TOP2A | 10563 |
| G2_M | TOP2B | 10564 |
| G2_M | TP53 | 10566 |
| G2_M | WEE1 | 11152 |
| G2_M | YWHAB | 11191 |
| G2_M | YWHAE | 11193 |
| G2_M | YWHAZ | 11195 |
| G2_M | CUL1 | 12551 |
| G2_M | PCAF | 12957 |
| G2_M | CCNB1 | 13010 |
| G2_M | BTRC | 13040 |
| G2_M | CCNB2 | 13228 |
| G2_M | CDC2 | 13897 |
| G2_M | CDC25B | 14004 |
| G2_M | CDC25C | 14014 |
| HIF | UBE2E3 | 423 |
| HIF | COPS5 | 831 |
| HIF | CREB1 | 1440 |
| HIF | CSNK1D | 1525 |
| HIF | NQO1 | 1837 |
| HIF | EDN1 | 1974 |
| HIF | EP300 | 2110 |
| HIF | EPO | 2137 |
| HIF | AKT1 | 2150 |
| HIF | ESR1 | 2163 |
| HIF | IKBKB | 2852 |
| HIF | UBE2S | 3859 |
| HIF | HIC1 | 4493 |
| HIF | HIF1A | 4494 |
| HIF | NR4A1 | 4553 |
| HIF | JUN | 5012 |
| HIF | LDHA | 5322 |
| HIF | LEP | 5329 |
| HIF | ARNT | 5449 |
| HIF | MDM2 | 5583 |
| HIF | ATM | 5968 |
| HIF | NOS2A | 6063 |
| HIF | NOS3 | 6064 |
| HIF | NPPA | 6086 |
| HIF | P4HB | 6207 |
| HIF | UBE2C | 6246 |
| HIF | UBE2J1 | 6649 |
| HIF | VEGFA | 7422 |
| HIF | HIF1AN | 7923 |
| HIF | TP53 | 10566 |
| HIF | UBE2A | 11011 |
| HIF | UBE2B | 11020 |
| HIF | UBE2D1 | 11029 |
| HIF | UBE2D2 | 11037 |
| HIF | UBE2D3 | 11040 |
| HIF | UBE2E1 | 11045 |
| HIF | UBE2G1 | 11051 |
| HIF | UBE2G2 | 11052 |
| HIF | UBE2H | 11053 |
| HIF | UBE2I | 11054 |
| HIF | UBE2L3 | 11056 |
| HIF | UBE2N | 11057 |
| HIF | SUMO1 | 11062 |
| HIF | VHL | 11126 |
| HIF | UBE2M | 13123 |
| HIF | UBE2L6 | 13354 |
| HIF | UBE2J2 | 118424 |
| JAK | AKT3 | 5 |
| JAK | CDKN1A | 237 |
| JAK | PIAS3 | 353 |
| JAK | CISH | 1187 |
| JAK | A2M | 2053 |
| JAK | AKT1 | 2150 |
| JAK | AKT2 | 2159 |
| JAK | FRAP1 | 3122 |
| JAK | GRB2 | 4098 |
| JAK | HRAS | 4634 |
| JAK | JAK1 | 5007 |
| JAK | JAK2 | 5008 |
| JAK | JAK3 | 5009 |
| JAK | KRAS | 5147 |
| JAK | MYC | 5871 |
| JAK | NRAS | 6102 |
| JAK | PIAS4 | 6715 |
| JAK | PIK3CA | 6912 |
| JAK | PIK3CB | 6913 |
| JAK | PIM1 | 6914 |
| JAK | PIK3CD | 6915 |
| JAK | PIK3R1 | 6917 |
| JAK | PIK3R2 | 6918 |
| JAK | MAPK1 | 8136 |
| JAK | MAPK3 | 8137 |
| JAK | MAP2K1 | 8164 |
| JAK | MAP2K2 | 8165 |
| JAK | PTPN1 | 8675 |
| JAK | PTPN6 | 8694 |
| JAK | PTPN11 | 8705 |
| JAK | RAF1 | 8843 |
| JAK | SHC1 | 9640 |
| JAK | STAT1 | 10264 |
| JAK | STAT2 | 10265 |
| JAK | STAT3 | 10266 |
| JAK | STAT4 | 10267 |
| JAK | STAT5A | 10268 |
| JAK | STAT5B | 10269 |
| JAK | STAT6 | 10270 |
| JAK | TIMP1 | 10494 |
| JAK | TYK2 | 10866 |
| JAK | PIK3R3 | 12644 |
| JAK | PIAS1 | 12712 |
| JAK | SOCS1 | 12792 |
| JAK | SOCS2 | 12943 |
| JAK | SOCS3 | 13099 |
| JAK | PIAS2 | 13150 |
| JAK | SOCS6 | 13406 |
| JAK | SOCS5 | 13727 |
| JAK | SOCS4 | 122809 |
| MAPK | RAPGEF3 | 363 |
| MAPK | YWHAQ | 819 |
| MAPK | YWHAQ | 819 |
| MAPK | CREB1 | 1440 |
| MAPK | CRK | 1456 |
| MAPK | PLCG1 | 1630 |
| MAPK | DUSP1 | 1936 |
| MAPK | DUSP2 | 1937 |
| MAPK | DUSP4 | 1939 |
| MAPK | DUSP6 | 1941 |
| MAPK | DUSP9 | 1945 |
| MAPK | EIF4E | 2034 |
| MAPK | EIF4EBP1 | 2035 |
| MAPK | ELK1 | 2059 |
| MAPK | ESR1 | 2163 |
| MAPK | ETS1 | 2175 |
| MAPK | ETS2 | 2176 |
| MAPK | PTK2B | 2236 |
| MAPK | FOS | 2959 |
| MAPK | FYN | 3156 |
| MAPK | MKNK2 | 4080 |
| MAPK | GRB2 | 4098 |
| MAPK | RAPGEF1 | 4102 |
| MAPK | H3F3A | 4418 |
| MAPK | H3F3B | 4419 |
| MAPK | HRAS | 4634 |
| MAPK | HSPB1 | 4673 |
| MAPK | HSPB2 | 4674 |
| MAPK | ARAF | 4982 |
| MAPK | KRAS | 5147 |
| MAPK | MOS | 5692 |
| MAPK | MYC | 5871 |
| MAPK | MYCN | 5873 |
| MAPK | ATF1 | 5912 |
| MAPK | NRAS | 6102 |
| MAPK | PAK1 | 6234 |
| MAPK | PIK3CA | 6912 |
| MAPK | PIK3CB | 6913 |
| MAPK | PIK3CD | 6915 |
| MAPK | PIK3R1 | 6917 |
| MAPK | PIK3R2 | 6918 |
| MAPK | PLA2G4A | 6940 |
| MAPK | PLCG2 | 6964 |
| MAPK | PPP1CA | 7446 |
| MAPK | PPP1CB | 7456 |
| MAPK | YWHAG | 7532 |
| MAPK | PRKACA | 7920 |
| MAPK | PRKACB | 7931 |
| MAPK | PRKACG | 7936 |
| MAPK | PRKAR1A | 7978 |
| MAPK | PRKAR2A | 8007 |
| MAPK | PRKAR2B | 8017 |
| MAPK | PRKCA | 8025 |
| MAPK | PRKCB1 | 8032 |
| MAPK | PRKCD | 8042 |
| MAPK | PRKCE | 8050 |
| MAPK | PRKCG | 8059 |
| MAPK | PRKCI | 8073 |
| MAPK | MAPK1 | 8136 |
| MAPK | MAPK3 | 8137 |
| MAPK | MAP2K1 | 8164 |
| MAPK | MAP2K2 | 8165 |
| MAPK | PTK2 | 8616 |
| MAPK | PXN | 8770 |
| MAPK | RAC1 | 8829 |
| MAPK | RAC2 | 8830 |
| MAPK | RAF1 | 8843 |
| MAPK | RAP1A | 8857 |
| MAPK | RPS6KA1 | 9117 |
| MAPK | BCAR1 | 9564 |
| MAPK | SHC1 | 9640 |
| MAPK | SOS1 | 10164 |
| MAPK | SOS2 | 10165 |
| MAPK | SRC | 10215 |
| MAPK | SRF | 10223 |
| MAPK | BRAF | 10229 |
| MAPK | STAT1 | 10264 |
| MAPK | STAT3 | 10266 |
| MAPK | TLN1 | 10511 |
| MAPK | YWHAB | 11191 |
| MAPK | YWHAH | 11194 |
| MAPK | YWHAH | 11194 |
| MAPK | YWHAZ | 11195 |
| MAPK | YWHAZ | 11195 |
| MAPK | TLN2 | 12415 |
| MAPK | PIK3R3 | 12644 |
| MAPK | MAPKAPK5 | 12709 |
| MAPK | MKNK1 | 12727 |
| MAPK | MAP2K1IP1 | 12789 |
| MAPK | RPS6KA4 | 13060 |
| MAPK | RPS6KA5 | 13361 |
| MAPK | KCNH8 | 131096 |
| MAPK | PPM1L | 151742 |
| MAPK | PPM1J | 333926 |
| metastasis | CDH6 | 35 |
| metastasis | CDH8 | 55 |
| metastasis | CDH11 | 79 |
| metastasis | CDH12 | 90 |
| metastasis | CDK4 | 166 |
| metastasis | CDKN2A | 266 |
| metastasis | GPNMB | 403 |
| metastasis | HTATIP2 | 490 |
| metastasis | HPSE | 721 |
| metastasis | METAP2 | 832 |
| metastasis | CHD4 | 913 |
| metastasis | PTP4A3 | 974 |
| metastasis | COL4A2 | 1334 |
| metastasis | CTBP1 | 1579 |
| metastasis | CTNNA1 | 1589 |
| metastasis | CTNNB1 | 1595 |
| metastasis | CTSK | 1625 |
| metastasis | CTSL | 1627 |
| metastasis | DCC | 1761 |
| metastasis | EPHB2 | 2126 |
| metastasis | ETV4 | 2180 |
| metastasis | ETV6 | 2183 |
| metastasis | EWSR1 | 2191 |
| metastasis | FAT | 2247 |
| metastasis | FGFR4 | 2344 |
| metastasis | FLT4 | 2726 |
| metastasis | FN1 | 2822 |
| metastasis | BRMS1 | 3291 |
| metastasis | GAPDH | 3355 |
| metastasis | TIAM2 | 3499 |
| metastasis | GNRH1 | 3931 |
| metastasis | GNRH2 | 3932 |
| metastasis | CDH19 | 4034 |
| metastasis | GZMA | 4398 |
| metastasis | HDAC1 | 4454 |
| metastasis | HGF | 4475 |
| metastasis | APC | 4622 |
| metastasis | HRAS | 4634 |
| metastasis | IGF1 | 4811 |
| metastasis | IL1B | 4865 |
| metastasis | IL8RB | 4889 |
| metastasis | IL18 | 4912 |
| metastasis | ITGA7 | 4972 |
| metastasis | ITGB3 | 4983 |
| metastasis | KISS1 | 5120 |
| metastasis | KRAS | 5147 |
| metastasis | RHOC | 5219 |
| metastasis | SMAD2 | 5487 |
| metastasis | SMAD4 | 5489 |
| metastasis | MCAM | 5563 |
| metastasis | MDM2 | 5583 |
| metastasis | MET | 5611 |
| metastasis | MGAT5 | 5623 |
| metastasis | TRPM1 | 5663 |
| metastasis | MMP2 | 5666 |
| metastasis | MMP3 | 5667 |
| metastasis | MMP7 | 5668 |
| metastasis | MMP9 | 5670 |
| metastasis | MMP10 | 5671 |
| metastasis | MMP11 | 5673 |
| metastasis | MMP13 | 5675 |
| metastasis | MYB | 5864 |
| metastasis | MYC | 5871 |
| metastasis | MYCL1 | 5872 |
| metastasis | NF2 | 6007 |
| metastasis | NME1 | 6053 |
| metastasis | NME2 | 6054 |
| metastasis | NME4 | 6056 |
| metastasis | PCOLN3 | 6462 |
| metastasis | SERPINB5 | 6891 |
| metastasis | PLAUR | 6947 |
| metastasis | FXYD5 | 7019 |
| metastasis | PNN | 7057 |
| metastasis | VEGFA | 7422 |
| metastasis | TMPRSS4 | 8304 |
| metastasis | B2M | 8320 |
| metastasis | PTEN | 8566 |
| metastasis | PTHLH | 8606 |
| metastasis | BAI1 | 8625 |
| metastasis | RB1 | 8875 |
| metastasis | ACTB | 8958 |
| metastasis | RORB | 9048 |
| metastasis | RPS27A | 9145 |
| metastasis | S100A4 | 9173 |
| metastasis | CCL7 | 9244 |
| metastasis | CXCL12 | 9274 |
| metastasis | GPR54 | 9291 |
| metastasis | SET | 9399 |
| metastasis | SLPI | 10076 |
| metastasis | SRC | 10215 |
| metastasis | SSTR2 | 10248 |
| metastasis | SYK | 10321 |
| metastasis | TCF20 | 10399 |
| metastasis | TGFB1 | 10462 |
| metastasis | TIAM1 | 10492 |
| metastasis | TIMP2 | 10495 |
| metastasis | TIMP3 | 10496 |
| metastasis | TIMP4 | 10497 |
| metastasis | TP53 | 10566 |
| metastasis | TPBG | 10571 |
| metastasis | TSHR | 10625 |
| metastasis | TWIST1 | 10808 |
| metastasis | VIL2 | 11128 |
| metastasis | CXCR4 | 11354 |
| metastasis | NR4A3 | 11948 |
| metastasis | RECK | 12524 |
| metastasis | CST7 | 12673 |
| metastasis | DENR | 12720 |
| metastasis | CAV1 | 12728 |
| metastasis | TNFSF10 | 12872 |
| metastasis | MTA1 | 13200 |
| metastasis | MTA2 | 13329 |
| metastasis | CD44 | 13679 |
| metastasis | MTSS1 | 13860 |
| metastasis | CDH1 | 14047 |
| NER | CDK7 | 198 |
| NER | ERCC8 | 1199 |
| NER | DDB1 | 1773 |
| NER | ERCC1 | 2147 |
| NER | ERCC2 | 2148 |
| NER | ERCC3 | 2152 |
| NER | ERCC4 | 2153 |
| NER | ERCC5 | 2154 |
| NER | ERCC6 | 2155 |
| NER | GTF2H1 | 4264 |
| NER | GTF2H2 | 4265 |
| NER | GTF2H3 | 4266 |
| NER | GTF2H4 | 4267 |
| NER | RPA4 | 4346 |
| NER | GTF2H5 | 5444 |
| NER | MNAT1 | 5684 |
| NER | POLR2A | 7084 |
| NER | POLR2B | 7085 |
| NER | POLR2C | 7086 |
| NER | POLR2D | 7089 |
| NER | POLR2E | 7091 |
| NER | POLR2F | 7095 |
| NER | POLR2G | 7096 |
| NER | POLR2H | 7100 |
| NER | POLR2I | 7101 |
| NER | POLR2J | 7103 |
| NER | POLR2K | 7104 |
| NER | POLR2L | 7106 |
| NER | POLR2J2 | 7345 |
| NER | RAD23B | 8836 |
| NER | RPA1 | 9055 |
| NER | RPA2 | 9056 |
| NER | RPA3 | 9057 |
| NER | XPA | 11176 |
| NER | XPC | 11177 |
| NER | CCNH | 13096 |
| NFkB | AKT3 | 5 |
| NFkB | EBI3 | 132 |
| NFkB | TLR6 | 307 |
| NFkB | MAP3K7IP1 | 400 |
| NFkB | MALT1 | 749 |
| NFkB | IRAK3 | 1025 |
| NFkB | CHUK | 1160 |
| NFkB | MAP3K8 | 1386 |
| NFkB | CREBBP | 1442 |
| NFkB | CSNK2A1 | 1531 |
| NFkB | CSNK2A2 | 1534 |
| NFkB | CSNK2B | 1536 |
| NFkB | EGF | 2003 |
| NFkB | EGFR | 2009 |
| NFkB | EP300 | 2110 |
| NFkB | AKT1 | 2150 |
| NFkB | AKT2 | 2159 |
| NFkB | IKBKB | 2852 |
| NFkB | GBP1 | 3548 |
| NFkB | GH1 | 3665 |
| NFkB | GHR | 3668 |
| NFkB | TRA@ | 4085 |
| NFkB | TBK1 | 4207 |
| NFkB | GSK3B | 4239 |
| NFkB | CARD10 | 4282 |
| NFkB | HDAC1 | 4454 |
| NFkB | HDAC2 | 4455 |
| NFkB | HRAS | 4634 |
| NFkB | HTR1A | 4691 |
| NFkB | IL1A | 4864 |
| NFkB | IL1B | 4865 |
| NFkB | IL1B | 4865 |
| NFkB | IL1R1 | 4866 |
| NFkB | IL1R1 | 4866 |
| NFkB | IL8 | 4886 |
| NFkB | INS | 4935 |
| NFkB | INSR | 4946 |
| NFkB | IRAK1 | 4952 |
| NFkB | AR | 4964 |
| NFkB | KRAS | 5147 |
| NFkB | UBE2V1 | 5176 |
| NFkB | LCK | 5316 |
| NFkB | LTA | 5448 |
| NFkB | LTBR | 5455 |
| NFkB | MAP3K3 | 5601 |
| NFkB | MYD88 | 5874 |
| NFkB | NFKB1 | 6020 |
| NFkB | NFKB1 | 6020 |
| NFkB | NFKB2 | 6021 |
| NFkB | NFKB2 | 6021 |
| NFkB | NFKBIA | 6022 |
| NFkB | NGFB | 6033 |
| NFkB | NGFR | 6034 |
| NFkB | NOS2A | 6063 |
| NFkB | NRAS | 6102 |
| NFkB | IRAK4 | 6421 |
| NFkB | TLR7 | 6517 |
| NFkB | TLR8 | 6544 |
| NFkB | TTRAP | 6704 |
| NFkB | PIK3CA | 6912 |
| NFkB | PIK3CB | 6913 |
| NFkB | PIK3CD | 6915 |
| NFkB | PIK3R1 | 6917 |
| NFkB | PIK3R2 | 6918 |
| NFkB | TRB@ | 6957 |
| NFkB | PLCG2 | 6964 |
| NFkB | PRKACA | 7920 |
| NFkB | PRKACB | 7931 |
| NFkB | PRKACG | 7936 |
| NFkB | PRKCB1 | 8032 |
| NFkB | PRKCQ | 8105 |
| NFkB | PRKCZ | 8116 |
| NFkB | MAPK8 | 8152 |
| NFkB | MAP2K6 | 8170 |
| NFkB | MAP2K7 | 8171 |
| NFkB | EIF2AK2 | 8175 |
| NFkB | CCND1 | 8912 |
| NFkB | RELA | 8929 |
| NFkB | RELB | 8930 |
| NFkB | BCL2L1 | 8937 |
| NFkB | TNFRSF17 | 9042 |
| NFkB | CCL5 | 9243 |
| NFkB | BMP2 | 9813 |
| NFkB | BMP4 | 9901 |
| NFkB | BMPR1A | 10057 |
| NFkB | BMPR1B | 10066 |
| NFkB | BMPR2 | 10075 |
| NFkB | BRCA2 | 10245 |
| NFkB | MAP3K7 | 10353 |
| NFkB | TGFA | 10461 |
| NFkB | TLR1 | 10513 |
| NFkB | TLR2 | 10514 |
| NFkB | TLR3 | 10515 |
| NFkB | TLR4 | 10516 |
| NFkB | TLR5 | 10519 |
| NFkB | TNF | 10539 |
| NFkB | TNFAIP3 | 10543 |
| NFkB | TNFRSF1A | 10546 |
| NFkB | TRAF2 | 10594 |
| NFkB | TRAF2 | 10594 |
| NFkB | TRAF3 | 10595 |
| NFkB | TRAF5 | 10596 |
| NFkB | TRAF5 | 10596 |
| NFkB | TRAF6 | 10597 |
| NFkB | TRAF6 | 10597 |
| NFkB | TNFSF13B | 10673 |
| NFkB | UBE2N | 11057 |
| NFkB | ZAP70 | 11196 |
| NFkB | IL1R2 | 11352 |
| NFkB | PIK3R3 | 12644 |
| NFkB | IKBKG | 12658 |
| NFkB | TNFSF11 | 12744 |
| NFkB | RIPK1 | 12865 |
| NFkB | TNFRSF11A | 12903 |
| NFkB | BCL10 | 13016 |
| NFkB | BTRC | 13040 |
| NFkB | MAP3K14 | 13097 |
| NFkB | MAP3K14 | 13097 |
| NFkB | CD40 | 13661 |
| NFkB | CD40LG | 13672 |
| NFkB | TLR9 | 54106 |
| NFkB | TLR10 | 81793 |
| NFkB | TIRAP | 114609 |
| NFkB | SFTPA1 | 653509 |
| p38 | MAP3K7IP1 | 400 |
| p38 | MAP4K1 | 1003 |
| p38 | DUSP10 | 1032 |
| p38 | CREB1 | 1440 |
| p38 | ATF2 | 1441 |
| p38 | MAPK14 | 1504 |
| p38 | DAXX | 1747 |
| p38 | DDIT3 | 1780 |
| p38 | DUSP1 | 1936 |
| p38 | ELK1 | 2059 |
| p38 | MKNK2 | 4080 |
| p38 | H3F3A | 4418 |
| p38 | H3F3B | 4419 |
| p38 | HMGN1 | 4542 |
| p38 | HSPB1 | 4673 |
| p38 | HSPB2 | 4674 |
| p38 | FAS | 4861 |
| p38 | IL1A | 4864 |
| p38 | IL1B | 4865 |
| p38 | IL1R1 | 4866 |
| p38 | FASLG | 4871 |
| p38 | MAX | 5549 |
| p38 | MEF2A | 5591 |
| p38 | MEF2B | 5592 |
| p38 | MEF2C | 5593 |
| p38 | MEF2D | 5594 |
| p38 | MAP3K5 | 5603 |
| p38 | MYC | 5871 |
| p38 | ATF1 | 5912 |
| p38 | MYEF2 | 6266 |
| p38 | PLA2G4A | 6940 |
| p38 | MAPK11 | 8154 |
| p38 | MAPK13 | 8161 |
| p38 | MAP2K3 | 8166 |
| p38 | MAP2K6 | 8170 |
| p38 | MAPK12 | 9196 |
| p38 | MAP2K4 | 9390 |
| p38 | SRF | 10223 |
| p38 | STAT1 | 10264 |
| p38 | MAP3K7 | 10353 |
| p38 | TGFB1 | 10462 |
| p38 | TGFB2 | 10464 |
| p38 | TGFB3 | 10465 |
| p38 | TGFBR1 | 10468 |
| p38 | TGFBR2 | 10470 |
| p38 | TNF | 10539 |
| p38 | TNFRSF1A | 10546 |
| p38 | TNFRSF1B | 10547 |
| p38 | TRAF2 | 10594 |
| p38 | TRAF6 | 10597 |
| p38 | IL1R2 | 11352 |
| p38 | MAPKAPK3 | 11360 |
| p38 | MAPKAPK5 | 12709 |
| p38 | MKNK1 | 12727 |
| p38 | TRADD | 12847 |
| p38 | FADD | 12889 |
| p38 | RPS6KA4 | 13060 |
| p38 | RPS6KA5 | 13361 |
| p38 | MAPKAPK2 | 13373 |
| p38 | EEF2K | 29904 |
| p38 | KCNH8 | 131096 |
| p53 | CDK4 | 166 |
| p53 | CDK7 | 198 |
| p53 | CDKN1A | 237 |
| p53 | CDKN2A | 266 |
| p53 | NDRG1 | 347 |
| p53 | TADA3L | 420 |
| p53 | PPP1R13L | 718 |
| p53 | CHEK1 | 935 |
| p53 | FAF1 | 946 |
| p53 | CHEK2 | 1019 |
| p53 | KLF6 | 1379 |
| p53 | DAPK1 | 1742 |
| p53 | DAXX | 1747 |
| p53 | GADD45A | 1779 |
| p53 | E2F1 | 1953 |
| p53 | E2F3 | 1956 |
| p53 | ESR1 | 2163 |
| p53 | FOXO3A | 2589 |
| p53 | PPP1R13B | 2839 |
| p53 | SIRT1 | 2874 |
| p53 | FRAP1 | 3122 |
| p53 | GAK | 3245 |
| p53 | GAPDH | 3355 |
| p53 | GAS1 | 3479 |
| p53 | AATF | 3603 |
| p53 | SESN1 | 3805 |
| p53 | GML | 3901 |
| p53 | SFN | 3947 |
| p53 | PYCARD | 4204 |
| p53 | HDAC1 | 4454 |
| p53 | HK2 | 4502 |
| p53 | APAF1 | 4556 |
| p53 | BIRC5 | 4675 |
| p53 | IFNB1 | 4791 |
| p53 | IGF1R | 4814 |
| p53 | CYR61 | 4831 |
| p53 | FASLG | 4871 |
| p53 | IL6 | 4880 |
| p53 | JUN | 5012 |
| p53 | KRAS | 5147 |
| p53 | PPP1R9B | 5508 |
| p53 | MCL1 | 5567 |
| p53 | MDM2 | 5583 |
| p53 | MDM4 | 5584 |
| p53 | MSH2 | 5803 |
| p53 | MYC | 5871 |
| p53 | MYOD1 | 5909 |
| p53 | ATM | 5968 |
| p53 | NF1 | 6005 |
| p53 | NFKB1 | 6020 |
| p53 | GTSE1 | 6676 |
| p53 | PHB | 6875 |
| p53 | PLAGL1 | 6943 |
| p53 | PLK1 | 6979 |
| p53 | PML | 7006 |
| p53 | PMP22 | 7010 |
| p53 | ATR | 7152 |
| p53 | LRDD | 7785 |
| p53 | PRKCA | 8025 |
| p53 | PRKCQ | 8105 |
| p53 | RPRM | 8276 |
| p53 | B2M | 8320 |
| p53 | PCBP4 | 8458 |
| p53 | PTEN | 8566 |
| p53 | BAG1 | 8570 |
| p53 | BAI1 | 8625 |
| p53 | BAK1 | 8701 |
| p53 | BAX | 8750 |
| p53 | RB1 | 8875 |
| p53 | BCL2 | 8920 |
| p53 | BCL2A1 | 8928 |
| p53 | RELA | 8929 |
| p53 | ACTB | 8958 |
| p53 | RPS27A | 9145 |
| p53 | BID | 9260 |
| p53 | P53AIP1 | 9320 |
| p53 | SHC1 | 9640 |
| p53 | SIAH1 | 9700 |
| p53 | SMARCB1 | 10083 |
| p53 | SNCA | 10129 |
| p53 | BNIP3 | 10146 |
| p53 | BRCA1 | 10220 |
| p53 | BRCA2 | 10245 |
| p53 | STAT1 | 10264 |
| p53 | TNF | 10539 |
| p53 | TP53 | 10566 |
| p53 | TP53BP2 | 10568 |
| p53 | TP73 | 10570 |
| p53 | TRAF2 | 10594 |
| p53 | TRAF5 | 10596 |
| p53 | TSC1 | 10620 |
| p53 | TSC2 | 10621 |
| p53 | TSHR | 10625 |
| p53 | WT1 | 11170 |
| p53 | BTG2 | 11343 |
| p53 | BAP1 | 12333 |
| p53 | CASP2 | 12393 |
| p53 | CASP9 | 12499 |
| p53 | PPM1D | 12625 |
| p53 | TP73L | 12767 |
| p53 | CRADD | 12866 |
| p53 | FADD | 12889 |
| p53 | TNFRSF10B | 12906 |
| p53 | PCAF | 12957 |
| p53 | CCNG2 | 13085 |
| p53 | CCNH | 13096 |
| p53 | CCNE2 | 13229 |
| p53 | PTTG1 | 13342 |
| p53 | EI24 | 13628 |
| p53 | TRAF4 | 13693 |
| p53 | CDC2 | 13897 |
| p53 | CDC25A | 13995 |
| p53 | CDC25C | 14014 |
| p53 | SESN2 | 83667 |
| p53 | SCGB3A1 | 92304 |
| p53 | SESN3 | 143686 |
